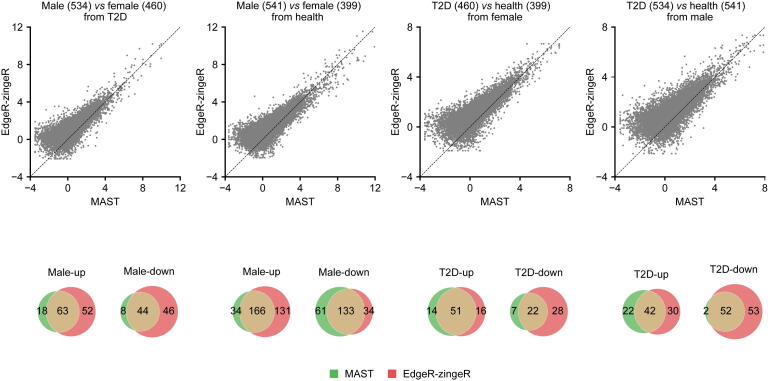# Single-cell RNA Sequencing Reveals Sexually Dimorphic Transcriptome and Type 2 Diabetes Genes in Mouse Islet β Cells

**DOI:** 10.1016/j.gpb.2021.07.004

**Published:** 2021-09-24

**Authors:** Gang Liu, Yana Li, Tengjiao Zhang, Mushan Li, Sheng Li, Qing He, Shuxin Liu, Minglu Xu, Tinghui Xiao, Zhen Shao, Weiyang Shi, Weida Li

**Affiliations:** 1 Translational Medical Center for Stem Cell Therapy and Institute for Regenerative Medicine, Shanghai East Hospital, Frontier Science Center for Stem Cell Research, School of Life Sciences and Technology, Tongji University, Shanghai 200092, China; 2 Tsingtao Advanced Research Institute, Tongji University, Qingdao 266073, China; 3 CAS Key Laboratory of Computational Biology, Shanghai Institute of Nutrition and Health, University of Chinese Academy of Sciences, Chinese Academy of Sciences, Shanghai 200031, China; 4 University of Chinese Academy of Sciences, Beijing 100049, China; 5 Department of Statistics, The Pennsylvania State University, University Park, PA 16802, USA; 6 Ministry of Education Key Laboratory of Marine Genetics and Breeding, College of Marine Life Sciences, Ocean University of China, Qingdao 266003, China

**Keywords:** Type 2 diabetes mellitus, Pancreatic β cell, Sex-biased gene expression, Sex-dependent T2D altered genes, Precision medicine

## Abstract

Type 2 diabetes (T2D) is characterized by the malfunction of **pancreatic β cells**. Susceptibility and pathogenesis of T2D can be affected by multiple factors, including sex differences. However, the mechanisms underlying sex differences in T2D susceptibility and pathogenesis remain unclear. Using single-cell RNA sequencing (scRNA-seq), we demonstrate the presence of sexually dimorphic transcriptomes in mouse β cells. Using a high-fat diet-induced T2D mouse model, we identified **sex-dependent T2D altered genes**, suggesting sex-based differences in the pathological mechanisms of T2D. Furthermore, based on islet transplantation experiments, we found that compared to mice with sex-matched islet transplants, sex-mismatched islet transplants in healthy mice showed down-regulation of genes involved in the longevity regulating pathway of β cells. Moreover, the diabetic mice with sex-mismatched islet transplants showed impaired glucose tolerance. These data suggest sexual dimorphism in T2D pathogenicity, indicating that sex should be considered when treating T2D. We hope that our findings could provide new insights for the development of **precision medicine** in T2D.

## Introduction

Currently type 2 diabetes (T2D) medication efficacy varies significantly among diabetic individuals, highlighting the importance of personalized treatment. However, a major challenge for precision medicine in T2D is the heterogeneous nature of the disease, including genetic predispositions (identified by genome-wide association studies), as well as sex, diet, and aging. Among these factors, sex differences provide an opportunity for personalized therapy, as sex is an easily recognizable trait.

However, in most current rodent metabolic studies, female animals are typically ignored. This is due to the fact that male animals tend to show more prominent disease phenotypes [1]. This experimental sex bias prevents a comprehensive understanding of the pathological mechanisms of metabolic disorders. As such, the National Institutes of Health (NIH) now demands that sex differences be reported in preclinical studies [2], [3]. Blood glucose homeostasis is controlled by the pancreatic islet, which is predominantly comprised of α cells, β cells, δ cells, and PP cells. α cells elevate blood glucose levels by secreting glucagon to promote hepatic glucose synthesis. β cells release insulin to decrease blood glucose levels by stimulating blood glucose uptake in fat, muscle, liver, and intestinal cells. δ cells secrete somatostatin to downregulate both α cells and β cell released hormones via paracrine signaling [4]. Polypeptides from PP cells, the rarest islet cell type, have effects on both gastric and pancreatic secretions [5].

Despite males and females having similar cellular compositions within islets, profound sex differences exist in islet physiological functions, metabolism, hormone-releasing signaling pathways, and diabetes occurrence [6]. For example, female rats are more susceptible than males to streptozotocin (STZ)-induced diabetes, suggesting that β cells of female rats are more sensitive to STZ toxicity than male β cells [6]. Similarly, maternal high-fat diet (HFD) results in insulin resistance and β cell loss due to oxidative stress, specifically in male offspring, but not in female offspring. This suggests that female islets may possess sex-specific protective mechanisms against oxidative stress [7]. In humans, T2D occurs more frequently in men with younger age and lower body mass index (BMI) than women [8], [9], [10]. Interestingly, whole islet genome-wide DNA methylation sequencing in humans shows insulin sexual dimorphism. Furthermore, islet methylome sex differences may be associated with differences in islet gene expression and insulin secretion levels [11]. However, sexual dimorphism of pancreatic islet β cells has not been investigated at the single-cell level. Such studies may reveal sex-specific gene expression differences in islet β cells and provide the basis for improved diabetes treatments for patients of both sexes.

Sex differences in diabetes susceptibility, development, and progression have been previously reported, suggesting the existence of sex-dependent diabetes-associated genes. Previous studies have shown that androgen receptor is specifically expressed in male islet β cells and plays an important role in regulating glucose-stimulated insulin secretion (GSIS) in both mice and humans [12]. *KLF14* allele variants also display increased female-specific T2D risk, likely due to female-specific fat storage and distribution [13]. Recent banding studies have reported that single-cell RNA sequencing (scRNA-seq) technology was applied to identify novel diabetes altered genes [14], [15], [16]. Nevertheless, the diversity of sex-dependent diabetes-associated genes and molecular pathways has not been comprehensively investigated from these studies.

Here, we systematically analyzed the single-cell gene expression profiles for β cells from healthy and diabetic mice. We find a considerable number of genes exhibiting sex-biased expression in β cells. Furthermore, we identify 62 sex-dependent diabetes-altered genes, suggesting that significant differences are present between males and females in the molecular mechanisms mediating diabetes pathogenesis. This conclusion was further supported by the sex-matched and sex-mismatched islet transplantation in mice. Compared to those in sex-matched transplants, genes involved in the longevity regulating pathway were down-regulated in β cells of sex-mismatched transplants. Concurrently, glucose tolerance notably decreased in diabetic mice transplanted with sex-mismatched islets. Given these results, we conclude that sex, as a biological variable, should be considered in diabetes treatment. Together, our results not only advance the current understanding of T2D pathogenesis, but also provide novel insights and targets for the development of sex-based precision medicine to treat T2D.

## Results

### Identification of β cell transcriptomes in male and female mouse pancreas

**Figure 1:**
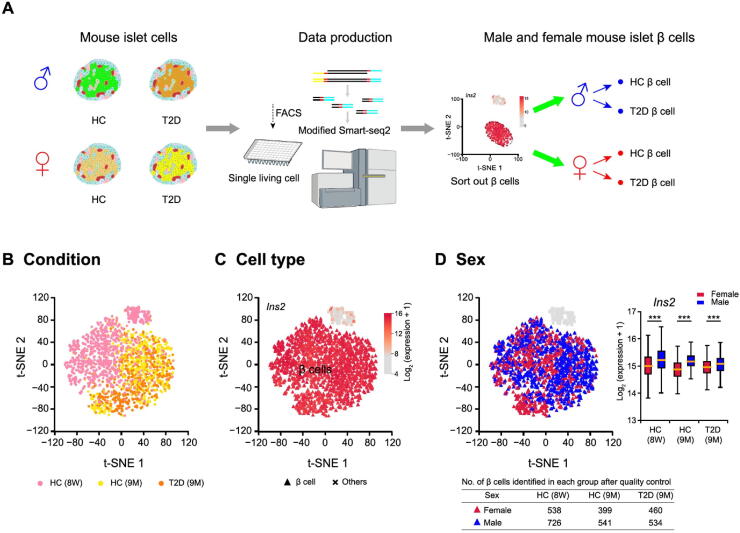
**Single islet β cell transcriptome analysis reveals sexually dimorphic genes in healthy and T2D mice** **A.** Schematic diagram for mouse single islet β cell RNA-seq data preparation and analysis. Mouse single living islet cells are labeled by Calcein Blue AM and collected through FACS. β cells are sorted out and divided into different groups according to donor conditions. **B.** t-SNE with sample information. The cells from HC and T2D mice are labeled with different colors, including 8-week-old HC (pink), 9-month-old HC (yellow), and 9-month-old T2D (orange) mice. **C.** t-SNE with cell type information. β cells are identified by the highly expressed marker gene *Ins2*, and are marked with triangles. **D.** t-SNE with sex information. The number of β cells identified in each group after quality control is listed in the table below. β cells from male mice are marked with blue triangles, and β cells from female mice are marked with red triangles. Significant difference in the expression of β cell marker gene *Ins2* between β cells of male and female mice is analyzed using Kolmogorov–Smirnov test. ***, *P* < 0.001. 8 W, 8-week-old; 9 M, 9-month-old; HC, healthy control; T2D, type 2 diabetes; FACS, fluorescence-activated cell sorting; t-SNE, t-distributed stochastic neighbor embedding.

**Supplementary figure S1:**
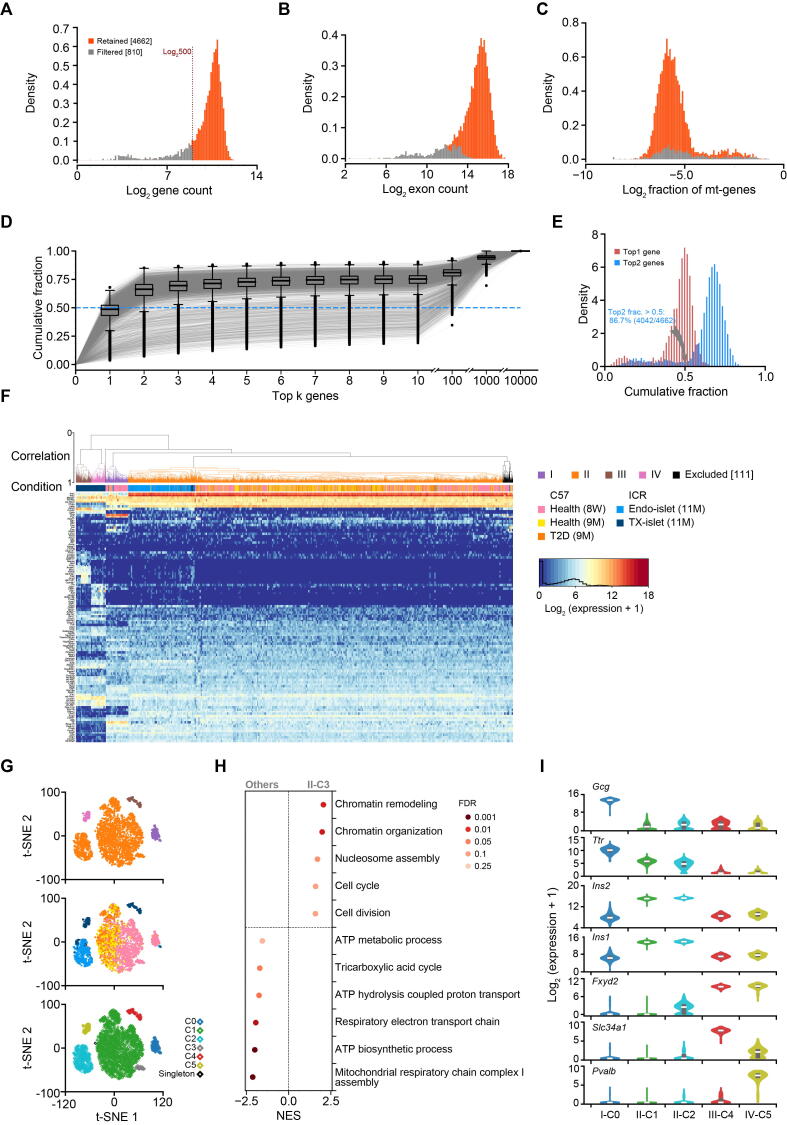


To obtain scRNA-seq profiles of mouse pancreatic β cells, we employed flow cytometry to isolate single cells from dissociated mouse islets via live dye staining (Figure 1A). In total, we collected and sequenced 5472 islet cells, with 3264 male cells and 2208 female cells. These included 1824 islet cells (1056 male and 768 female) from 8-week-old healthy mice, 2208 islet cells (1152 male and 1056 female) from 9-month-old healthy and diabetic mice, 672 male transplanted islet cells (9 months post-transplantation) in kidney capsules from both male and female recipient mice, and 768 endogenous pancreatic islet cells (384 male and 384 female) from recipient mice (detailed sample information in Table 1). scRNA-seq libraries were constructed using a modified Smart-seq2 protocol. The average library size was 370,000 reads per cell, and the average number of genes detected per cell was about 1500. We retained 4662 cells that had at least 500 genes detected (Figure S1A). These retained cells had high total unique molecular identifier (UMI) counts mapped to gene exons (Figure S1B), and low fractions of UMI counts of mitochondrial genes (Figure S1C). These data suggest that we obtained high-quality scRNA-seq profiles for mouse islets.

**Table 1:** **No. of islet cells collected from mice under different conditions for scRNA-seq library construction**

Strain	Age	Group	No. of collected islet cells		Subtotal
			Male	Female	
C57BL/6J	8 W	HC	1056	768	1824
C57BL/6J	9 M	T2D	576	576	1152
C57BL/6J	9 M	HC	576	480	1056
ICR	11 M	Endogenous	384	384	768
ICR	11 M	Transplanted	288	384	672
Total			2880	2592	5472

*Note*: Cells in “endogenous” group include islet cells that were isolated from pancreas of male and female recipient mice. Cells in “transplanted” group were dissected from the kidney capsule of recipient mice, including sex-matched (male to male) and sex-mismatched (male to female) islet transplants at the time point of 9 months post-transplantation. 8 W, 8-week-old; 9 M, 9-month-old; 11 M, 11-month-old; HC, healthy control; T2D, type 2 diabetes.

From these 4662 cells, we next identified β cells for downstream analysis. Firstly, we applied an adjusted count per million (adjCPM) method to normalize our scRNA-seq data by excluding the union of the top 2 genes with the highest expression from all cells while calculating the normalization factors (Figure S1D and E; see Materials and methods). Secondly, we performed hierarchal clustering of scRNA-seq profiles based on the union of the top 10 highly expressed genes from all cells, and 111 cells were excluded (Figure S1F). This clustering was subsequently used to determine cellular identities. We then performed principal component analysis (PCA) of identified hypervariable genes (HVGs), with results being visualized using t-distributed stochastic neighbor embedding (t-SNE) plots (Figure S1G; see Materials and methods). To identify the sexually dimorphic genes, 3420 cells from healthy (8-week-old and 9-month-old) and diabetic (9-month-old) C57BL/6J mice were selected for analysis. We found that islet cells from healthy (8-week-old and 9-month-old) and diabetic mice were aggregated into two distinct clusters and exhibited differential expression of the β cell marker gene *Ins2* (Figure 1B and C). Among the 3420 cells, we identified 3198 β cells (1801 male and 1397 female), as defined by their high *Ins2* expression (Figure 1C, Figure S2A). The mouse β cell sex of identified is provided on the t-SNE map (Figure 1D) and was further confirmed by the expression of X and Y chromosome genes (Figure S2B). Interestingly, we found that male and female β cell profiles did not completely overlap (Figure 1D). To further investigate potential sexually dimorphic genes, we applied a Kolmogorov–Smirnov test to determine the differential expression of *Ins2* between male and female β cells. We found a significant difference in *Ins2* expression between male and female β cells (Figure 1D), suggesting β cell transcriptome sexual dimorphism.

### Sex-biased gene expression in mouse β cells under healthy and T2D conditions

**Figure 2:**
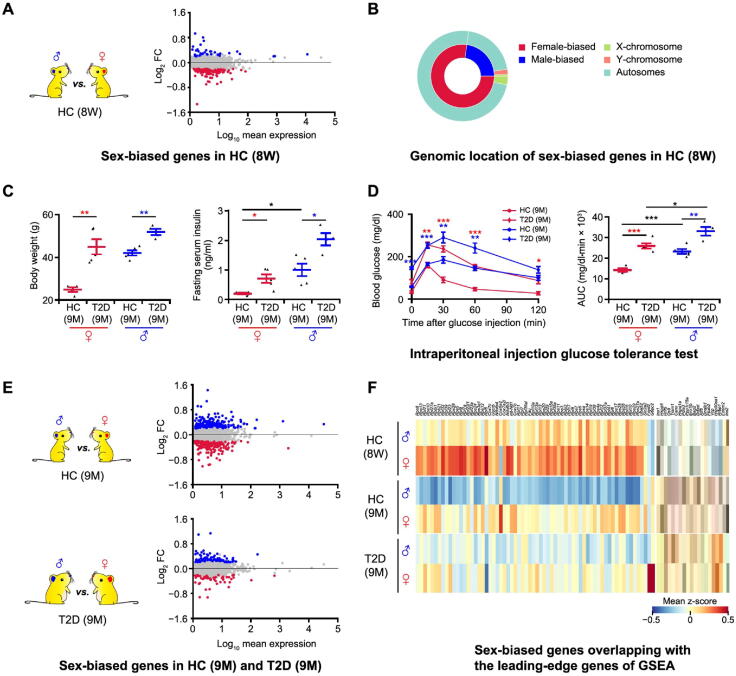
**Sex-biased genes exist in both healthy and T2D mice** **A.** Differential gene expression analysis between β cells in 8-week-old male and female HC C57BL/6J mice. In the MA plot, the male-biased and female-biased genes are indicated in blue and red, respectively. Gene expression levels are calculated as log_2_ normalized UMI counts and the obtained vaues are further log_10_ tranformed for easy view in the plot. More details are provided in Table S1. **B.** Nested pie chart depicting the genomic location of sex-biased genes. **C.** Diabetes-associated physiological phenotypes. Body weight and serum insulin level are detected after 6 h fasting. The blue dots represent the male HC (*n* = 5) and T2D mice (*n* = 4); the red dots represent the female HC (*n* = 4) and T2D (*n* = 5) mice. Data are presented as mean ± SEM. *, *P* < 0.05; **, *P* < 0.01 (two-sample *t*-test). **D.** Intraperitoneal injection glucose tolerance test. Time course is shown on the left and AUC values are shown on the right. The blue lines represent the male HC (*n* = 5) and T2D mice (*n* = 4); the red lines represent the female HC (*n* = 4) and T2D (*n* = 5) mice. Red asterisks represent female T2D *vs.* female HC mice; blue asterisks represent male T2D *vs.* male HC mice. Data are presented as mean ± SEM. *, *P* < 0.05; **, *P* < 0.01; ***, *P* < 0.001 (two-sample *t*-test). **E.** Differential gene expression analysis between β cells in 9-month-old male and female HC and T2D mice. In the MA plots, the male-biased and female-biased genes are indicated in blue and red, respectively. More details are provided in Tables S2 and S3. **F.** Heatmap showing sex-biased genes overlapping with the leading-edge genes of GSEA. The color scale shows the mean value of z-score, with blue and red corresponding to the minimum and maximum values of standardized log_2_ (expression + 1), respectively. More details are provided in Table S4. DEG, differentially expressed gene; GSEA, gene set enrichment analysis; AUC, area under curve.

To identify genes that display sex-biased expression patterns in mouse pancreatic β cells, we first compared β cell transcriptional profiles of healthy male and female 8-week-old C57BL/6J mice (Figure 2**A**). Differentially expressed genes (DEGs) were identified using model-based analysis of single-cell transcriptomics (MAST) [17], with a false discovery rate (FDR) cutoff of 0.05. This FDR cutoff was deployed for all MAST-based differential expression analyses. In total, we identified 162 DEGs, with 37 genes displaying higher expression in males and 125 genes displaying higher expression in females (Figure 2A; Table S1). Among these, only 4 genes are located on sex chromosomes (*Xist* on the X chromosome; *Eif2s3y*, *Ddx3y*, and *Uty* on the Y chromosome) (Figure 2B). These data suggest that significant sexual dimorphism exists in β cell transcriptomes of healthy mice.

Next, we investigated whether β cell transcriptome sexual dimorphism also exists under diabetic conditions. To investigate this, we performed scRNA-seq on β cells from HFD-induced diabetic mice. These C57BL/6J mice are fed with HFD starting at 8 weeks old to establish a T2D mouse model with impaired β cell function [18]. Expected T2D phenotypes were observed in both male and female diabetic mice, including high body weight, increased fasting insulin levels, and impaired glucose tolerance (Figure 2C and D). Notably, we also observed sex-associated differences in fasting serum insulin levels of healthy mice. Specifically, the fasting serum insulin level of healthy male mice was significantly higher than that of healthy female mice (Figure 2C). In addition, the area under curve (AUC) of the glucose tolerance test showed that there were sex-associated differences in glucose tolerance between males and females in both healthy and T2D mice (Figure 2D). To investigate the mechanisms underlying these observed differences, we performed single β cell transcriptome sequencing on 9-month-old T2D mice (HFD feeding up to 7 months) and age-matched healthy mice (normal diet, ND) of both sexes. MAST was used to determine transcriptional profile differences across these experimental conditions. We identified 394 DEGs between male and female in 9-month-old healthy mice, including 200 genes with higher expression in male β cells and 194 genes with higher expression in female β cells (Figure 2E; Table S2). In T2D mice, we also identified 81 genes with higher expression in male β cells and 52 genes with higher expression in female β cells (Figure 2E; Table S3).

To elucidate potential sex-biased pathways based on the aforementioned results, gene set enrichment analysis (GSEA) was performed with FDR < 0.25. This FDR cutoff was used for all GSEA analyses [19]. In β cells from both 8-week-old and 9-month-old healthy animals, the longevity-regulating pathway was enriched in males (Figure S2C and D); for pathway-related genes, *Ins1* was expressed significantly higher in 8-week-old healthy males, *Ins2* and *Hspa1a* were expressed significantly higher in 9-month-old healthy males, and *Hspa8* was expressed significantly higher in both 8-week-old and 9-month-old healthy males (Tables S1 and S2). Intriguingly, the ribosome pathway was consistently enriched in female β cells from both healthy (8-week-old and 9-month-old) and diabetic mice (Figure S2C–E). Importantly, in β cells from diabetic mice, both the *N*-glycan biosynthesis pathway and Notch signaling pathway were enriched in males (Figure S2E). Conversely, the JAK-STAT signaling pathway, ferroptosis pathway, spliceosome pathway, as well as carbohydrate digestion and absorption pathway were enriched in female β cells from diabetic mice (Figure S2E). The sex-biased genes included in the leading-edge genes of enriched pathways are shown in the heatmap (Figure 2F; Table S4). In summary, our results validated the presence of sex-biased gene expression and pathways in β cells from both healthy and diabetic mice, indicating that the pathological mechanisms of T2D may differ between males and females.

### Extensive sex-dependent T2D gene expression alterations in mouse β cells

**Figure 3:**
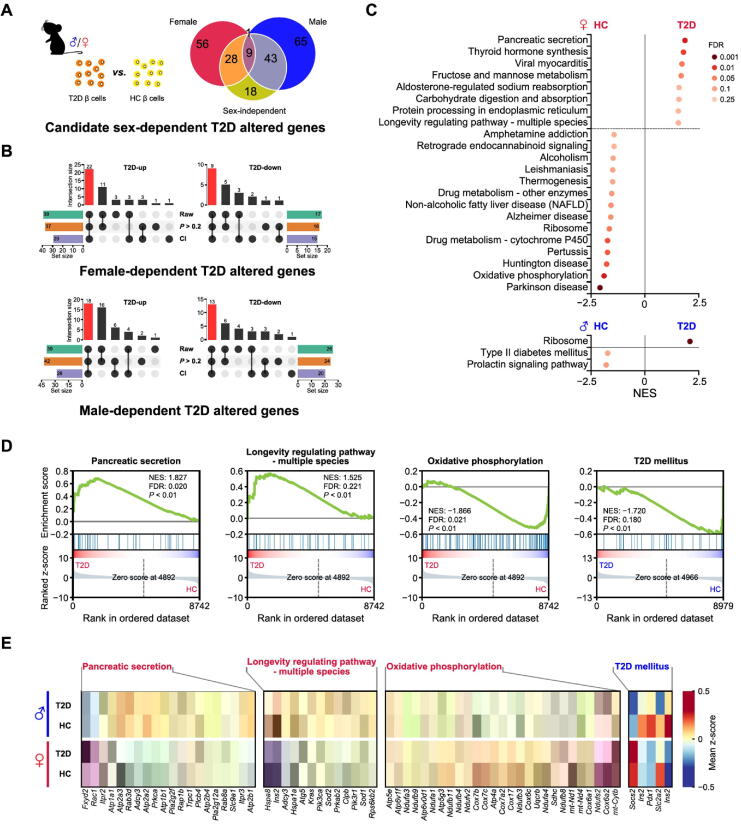
**T2D altered genes and pathways differ in a sex-dependent manner** **A.** Venn diagram depicting the definition of potential sex-dependent T2D altered genes. **B.** Upset plot shows the definition of sex-dependent T2D altered genes. The red bar shows the number of sex-dependent T2D altered genes in each group. “Raw” represents the candidate genes defined in Figure 3A. “CI” represents the genes whose CI of log_2_ FC from analysis of one sex does not overlap with that from the opposite sex. “*P* > 0.2” represents that the *P* values in the non-significant sex group are high. The bar chart at bottom left or right represents the number of genes under each filter condition. The bar chart above the dot plot represents the number of genes in each group that fit different filter condition (black dot). More details are provided in Table S6. **C.** Results of GSEA showing pathways significantly enriched in the HC group and the T2D group in female (labeled in red) and male (labeled in blue) mice. Pathways with NES > 0 are enriched in β cells of T2D mice, and pathways with NES < 0 are enriched in β cells of HC mice. **D.** GSEA plots of pathways involved in the onset of T2D or the function of β cells. **E.** Heatmap showing leading-edge genes of GSEA included in the selected pathways. The pathway in red represents the female-specific enriched pathway in β cells of T2D or HC mice. The pathway in blue represents male-specific enriched pathway in β cells of HC mice. T2D-up, T2D up-regulated genes; T2D-down, T2D down-regulated genes; CI, confidence interval; NES, normalized enrichment score.

Given our findings of sex-biased gene expression differences in β cells of T2D mice, we hypothesized that T2D development may differ in males and females. Previous studies have used single-cell transcriptional profiles of islets from T2D mice to identify T2D altered genes. However, these studies did not consider the factor of sex [14], [15], [16]. To dissect the role of sex in these processes, we compared the single-cell transcriptome of β cells from T2D mice with that from age-matched healthy mice. First, we used MAST to perform differential expression analysis between β cells of T2D and healthy mice of both sexes. Both cellular detection rate (CDR) and sex were provided as covariates, and we identified 98 DEGs (40 down-regulated and 58 up-regulated) as sex-independent T2D altered genes (Table S5). We next compared β cells of T2D mice with β cells of healthy mice of the same sex to obtain DEGs in a male- or female-specific manner. These DEGs of each sex were then matched against the 98 sex-independent genes mentioned earlier. The non-overlapping genes in this set were defined as potential sex-dependent T2D altered genes. In total, we identified 56 and 65 genes in female and male β cells, respectively (Figure 3**A**).

To further refine our sex-dependent T2D candidate genes, we introduced two additional filtering criteria: 1) the estimated 95% confidence interval of log_2_ fold change (log_2_ FC) by MAST from the differential expression analysis in one sex should not overlap with that from the other sex, and 2) the *P* value in the non-significant sex group was higher than 0.2. We selected the genes passing both of these two additional criteria as the final sex-dependent T2D altered genes. As a result, we identified 31 female-specific (22 up-regulated and 9 down-regulated genes in T2D, as compared to healthy controls) and 31 male-specific (18 up-regulated and 13 down-regulated genes in T2D, as compared to healthy controls) T2D altered genes (Figure 3B; Table S6). We further validated 14 sex-dependent T2D altered genes with baseMean > 10 by qRT-PCR assay (Figure S3A).

Among these female-dependent T2D altered genes, we found up-regulation of both *Serp1* and *Ero1lb*. Interestingly, both of these genes encode endoplasmic reticulum (ER) stress-associated proteins. Furthermore, overexpression of *Ero1lb* in mouse β cells leads to up-regulation of unfolding protein-associated genes and ER stress [20]. The intervention of conjugated estrogens could protect postmenopausal women from diabetes by promoting the degradation of misfolded proteins during insulin synthesis in β cells [21]. Taken together, our findings suggest that sex-dependent T2D differences may be due to differences in ER stress responses.

Among male-dependent T2D altered genes, several genes were reported to confer sex-specific effects on β cell function or affect the onset of T2D. Notably, our analysis identified *Iapp* as an up-regulated male-dependent T2D gene. Accumulation of Iapp protein has previously been shown to be toxic to pancreatic β cells [22]. This previous study found that ectopic expression of human *IAPP* in the pancreatic β cells of transgenic mice led to a more severe diabetic phenotype in males but not females, suggesting sex-dependent pathogenesis. We also identified two mitochondrial function-related genes, *Cox4i1* and *Ndufb7*, which were down-regulated in β cells of male T2D mice. Interestingly, insulin secretion capacity is associated with mitochondrial function and declines with age, as it has been reported that elderly male Wistar rats show more significantly decreased mitochondrial function and insulin secretion than elderly females [23]. Put together, our findings suggest that sex-dependent mitochondrial function may also play a key role in the pathogenesis of T2D.

For the remaining sex-dependent T2D altered genes we identified, few reports exist regarding sex-dependent expression. These genes include *Cpe*, *Scg3*, *Ttr*, *Rnase4*, *Malat1*, *Ucn3*, *Pura*, and *Tmed3*. As female mice are typically discarded in such studies, little is known about the role of certain sex-dependent genes in T2D pathogenesis [1]. These genes are compelling candidates for future studies into sex-associated differences in T2D pathogenesis.

To further investigate sex differences in T2D altered pathways, we performed GSEA by comparing β cell transcriptomes from T2D and healthy mice of each sex. In females, several pathways enriched in β cells of T2D mice, such as the pancreatic secretion pathway and longevity regulating pathway (multiple species), had direct links to β cell function and the onset of T2D. Conversely, the oxidative phosphorylation pathway and ribosome pathway were enriched in β cells of healthy mice. At the same time, in males, β cells of T2D mice had ribosome pathway enriched, and β cells of healthy mice had T2D mellitus pathway enriched (Figure 3C and D). The expression patterns of genes involved in the pathways mentioned above were shown in the heatmap (Figure 3E) and violin plots (Figure S3B). Together, both the distinctive patterns of sex-dependent T2D altered genes and the different T2D-associated pathways suggest a sex-dependent effect in T2D pathogenesis.

Although we detected no common gene set that showed significant enrichment in both sexes, we found numerous sex-independent DEGs (98 in total; 40 down-regulated and 58 up-regulated) (Table S5) that are involved in fundamental β cell function or T2D pathogenesis. For example, we found that the insulin secretion regulating genes *Slc2a2*, *Calm2*, and *c-Fos* are down-regulated in a sex-independent manner. *Slc2a2* encodes the glucose transporter 2 (Glut-2). Glut-2 functions as a membrane protein to transport glucose into β cells to mediate GSIS, which is crucial for glucose homeostasis [24]. Previous evidence has shown that mutations in *Slc2a2* result in neonatal diabetes in both male and female patients [25]. Moreover, pathogenic *Slc2a2* variants are also associated with an increased risk for the transition from impaired glucose tolerance to T2D [26]. *Calm2* encodes the Camodule-2 protein and mediates calcium signaling in GSIS [27]. Overexpression of *c-Fos* in β cells contributes to increased insulin secretion, whereas *c-Fos* knockdown results in decreased insulin secretion [28]. Together, these results indicate that insulin secretion impairment may be a common pathological mechanism for T2D in both males and females.

Lastly, we also observed sex-independent up-regulated genes involved in many critical cellular events of T2D pathogenesis, including transdifferentiation and ER stress. As reported previously, β-to-α cell (glucagon producing) transdifferentiation occurs in T2D pathogenesis [29], [30]. Our results are consistent with this finding with *Gcg* (encoding glucagon) being an up-regulated sex-independent T2D gene. *Hsp90aa1* was also up-regulated in β cells of both male and female T2D mice. Previous studies have indicated Hsp90 (encoded by *Hsp90aa1*) as a therapeutic target for ER stress in T2D. Inhibition of Hsp90 alleviates insulin resistance and improves glucose tolerance [31], [32]. Collectively, these results suggest the presence of common T2D pathogenesis mechanisms between sexes.

### Mice with sex-matched β cell transplants show improved glucose homeostasis

**Figure 4:**
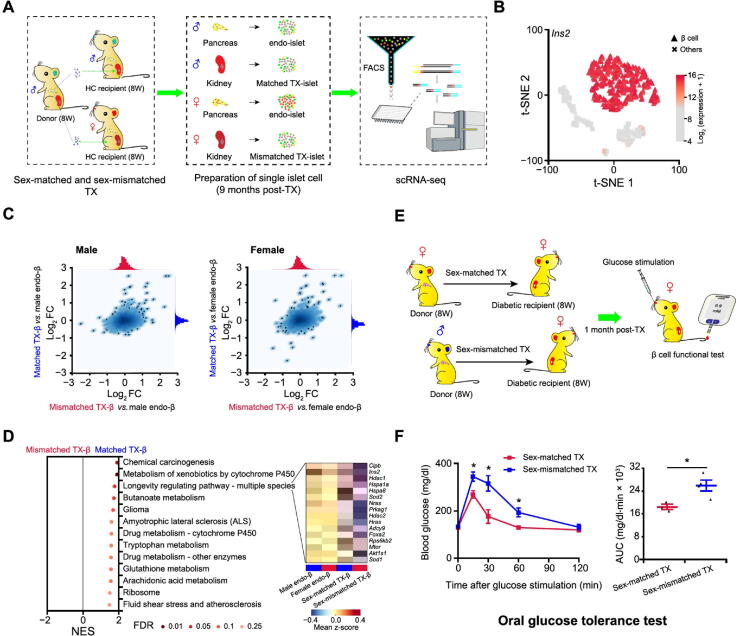
**Sex-matched islet TX confers better glucose tolerance than sex-mismatched transplantation** **A.** Schematic diagram of sex-matched and sex-mismatched islet TX for scRNA-seq. Single islet cells are collected for scRNA-seq 9 months post-TX. **B.** t-SNE with cell type information. β cells from endo-islet and TX-islet cells in both sex-matched and sex-mismatched TX experiments are identified by high expression of *Ins2*. More details are provided in Figure S2A. **C.** Correlation analysis between TX-β and endo-β cells. Scatterplot depicting correlation of log_2_ FC values generated by comparing TX-β cells (sex-matched or sex-mismatched) to male endo-β cells (left panel) or female endo-β cells (right panel). **D.** GSEA of sex-matched and sex-mismatched TX-β cells. Leading-edge genes of the longevity regulating pathway are zoomed in with heatmap. **E.** Schematic diagram of sex-matched and sex-mismatched islet TX for β cell functional test in STZ-induced female diabetic mice. **F.** Oral glucose tolerance test for mice after sex-matched and sex-mismatched islet TX. Time course is shown on the left and AUC values are shown on the right. Glucose (2 g/kg body weight) is gavaged after 6 h fasting, and blood glucose level is detected at 0 min, 15 min, 30 min, 60 min, and 120 min after glucose gavage. The blue line represents the sex-mismatched islet TX (*n* = 4); the red line represents sex-matched islet TX (*n* = 3). Data are presented as mean ± SEM. *, *P* < 0.05 (two-sample *t*-test). TX, transplanted/transplantation; endo-islet, endogenous islet of recipient mouse; endo-β, endogenous β cell of recipient mouse; TX-β, transplanted β cells; STZ, streptozotocin.

The existence of sexual dimorphism in mouse β cell transcriptomes suggests that sex is an important factor in β cell function and pathogenesis of T2D and should be emphasized when treating diabetes. To validate this critical concept, we performed long-term islet transplantations (an experimental treatment for insulin insufficient diabetes mellitus) with sex-matched and sex-mismatched islets in ICR mice. ICR mice were selected as donors and recipients in this assay as they do not develop spontaneous insulitis and diabetes. Therefore, they are more suitable than C57BL/6 for assessing the long-term effects of islet transplantation. Islets from 8-week-old male mice were isolated and transplanted into kidney capsules of age-matched male or female mice. Transplanted islets (TX-islets) and endogenous islets (endo-islets) from recipient mice were all collected at 9 months post-transplantation and dissociated for scRNA-seq profiling (Figure 4**A**). A total of 714 β cells were identified by high-level expression of *Ins2* as previously described (Figure 4B, Figure S2A). Differential gene expression analysis in β cells was performed in two ways. Firstly, we plotted the gene expression changes observed by comparing the transcriptomes of sex-matched and sex-mismatched transplanted β cells to that of male (Figure 4C, left panel) or female (Figure 4C, right panel) endogenous β cells. In both analyses, we observed a clear correlation between the gene expression changes detected from the two comparisons. This indicated that most of the DEGs showed consistent expression changes between transplanted and endogenous β cells in male and female recipients. However, it can also be observed that the distribution of the log_2_ FC values shown on the x-axis was clearly wider than the distribution of those shown on the y-axis in both plots. This suggests that the overall transcriptome of the sex-matched transplanted β cells was closer to that of endogenous β cells (for both male and female recipients), than the overall transcriptome of the sex-mismatched transplanted β cells. Secondly, differential gene expression analysis was performed between the transcriptomes of sex-matched and sex-mismatched transplanted β cells. Compared to the sex-mismatched group, sex-matched transplanted β cells had 3 genes (*Kap*, *Sfrp5*, and *Akr1c21*) significantly up-regulated and 2 genes (*Ovol2* and *Matn2*) significantly down-regulated (Figure S3C and D). Notably, *Sfrp5* was a male-biased expressed gene in β cells from both 8-week-old and 9-month-old healthy mice (Tables S1 and S2). Previous studies have shown that overexpression of *Sfrp5*, which is down-regulated in obesity and T2D, can ameliorate impaired glucose tolerance in mice [33]. In humans, higher serum SFRP5 levels are correlated with a lower risk of T2D onset [34]. Moreover, our GSEA results showed that a longevity regulating pathway was significantly enriched in sex-matched transplanted β cells. Among the 16 leading-edge genes, *Ins2*, *Sod1*, *Sod2*, and *Foxa2* are directly related to β cell insulin secretion (Figure 4D). Collectively, these results suggest that sex-matched islet transplantation could be more beneficial to the function of β cells of the transplants, thus contributing to better control of glucose homeostasis.

To further define the potential benefits of sex-matched islet transplantation, we transplanted islets from male or female mice into STZ-induced female diabetic mice (Figure 4E). Hyperglycemia (>350 mg/dl) of the diabetic recipient with sex-matched or sex-mismatched transplantation was restored to normal levels 1 month post-transplantation (<144 mg/dl; Figure S3E). Interestingly, the glucose tolerance of the diabetic mice with sex-matched transplantation was significantly better, as measured by an oral glucose tolerance test (Figure 4F). In summary, our transcriptomic characterization of β cells in diabetic and healthy male and female mice, as well as our transplantation studies, suggests that sex should be considered in diabetes treatment.

## Discussion

Previous studies have shown that sexual dimorphism exists in many organs and body systems, including the heart, the kidney, the immune system, and the central nervous system [35], [36], [37], [38]. T2D is a complex metabolic disorder characterized by islet β cell failure and is impacted by sex. Sex differences in islet β cell physiological function and diabetes prevalence have been previously recognized; however, to date, there has been no in-depth transcriptomic investigation of sex differences in T2D. To decipher potential sexual dimorphism in T2D pathogenesis, we used scRNA-seq to comprehensively characterize the transcriptomes of β cells in healthy and T2D mice in both male and female mice. We identified numerous genes that displayed significant sex-biased expression in β cells of both healthy and T2D mice. Furthermore, we identified 62 sex-dependent T2D altered genes in mice, suggesting that sex-based differences may impact the molecular mechanisms of diabetes pathogenesis in T2D mouse model. Collectively, our results provide potentially innovative sex-specific therapies for the precision medicine treatment of T2D.

Interestingly, we found that the longevity regulating pathway was enriched specifically in β cells of healthy male mice. Previous evidence has shown that insulin and insulin-like growth factor signaling are involved in the longevity pathway, suggesting a crucial role for metabolic glucose homeostasis in aging and life span [39], [40], [41]. Furthermore, expression levels of the insulin-coding genes, *Ins1* and *Ins2*, were significantly up-regulated in β cells of 8-week-old and 9-month-old healthy male mice, respectively, suggesting that these mice have enhanced insulin synthesis. Similarly, the longevity pathway-related genes *Hspa8* (in β cells of 8-week-old and 9-month-old healthy mice) and *Hspa1a* (in β cells of 9-month-old healthy mice) showed significant up-regulation in male mouse β cells. Both *Hspa1a* and *Hspa8* have been shown to protect β cells from oxidative stress [42], [43]. Up-regulation of *Hspa1a* is also associated with extended longevity [42], suggesting that healthy male mice have mechanisms to protect against the stress caused by increased insulin synthesis.

Currently, islet transplantation clinical trials do not emphasize sex matching between donors and recipients. From our work here, we conclude that sex is a crucial biological variable that should be considered in the treatment of diabetes. This conclusion is further supported by our sex-matched and sex-mismatched islet transplantation experiments in mice. Indeed, compared with the sex-mismatched group, the β cells of transplants in the sex-matched group showed significant enrichment of the longevity regulating pathway (consistent with the results shown in Figure S2C and D), with up-regulated expression of pathway-related genes, including *Ins2*, *Hspa1a*, and *Hspa8*, as well as *Sod1*, *Sod2*, and *Hdac1* (three genes involved in the extension of the organism longevity) [44], [45], [46]. The long-term curative effect of islet transplantation is impacted by transplant survival [47]. Moreover, the glucose tolerance of the sex-mismatched transplanted mice was notably improved. Taken together, our results suggest that sex should be considered during islet transplantation for achieving the long-term stability and function of islet transplants.

T2D susceptibility is also affected by sex-based differences in sex steroid hormones. Endogenous estrogens are protective against T2D in females, and T2D risk increases following menopause, which is associated with decreased estrogen [48], [49]. Estrogen not only improves islet β cell function and survival [50], but also stimulates the secretion of GLP-1 from islet α cells and intestine L cells to maintain glucose homeostasis [51]. Conversely, testosterone, the male sex hormone, increases the T2D risk in males [52], [53]. A recent report has revealed that testosterone improves insulin secretion through androgen receptor on male islet β cells in both mice and humans [12]. Taken together, sex differences in both islet β cells and sex steroid hormones need to be further investigated for the development of sex-specific precision medicine and T2D treatment.

## Materials and methods

### Animals and HFD-induced diabetic mice

We housed all the mice under the specific pathogen-free grade environment of the animal facility at Tongji University, Shanghai, China. Adult male and female mice (8 weeks old) were purchased from Shanghai Slac Laboratory Animal. To establish mouse T2D model, male and female C57BL/6J mice (*n* = 5 per sex) were fed with HFD from 8 weeks old to 9 months old. These mice were included as the T2D mice, and an equal number of age-matched male and female mice fed with ND were included as healthy control mice.

### Islet transplantation

Islets were isolated from 8-week-old male ICR mice (*n* = 20), and the detailed process of islet isolation was previously described [54]. Similar size islets were handpicked after purification under a stereomicroscope, and three male and three female age-matched healthy ICR mice were selected as recipients with about 300–400 islets being transplanted under the kidney capsules. About 9 months after transplantation, TX-islets were dissected and scraped from the kidney capsules, and the pellet was collected and dissociated into single cells for scRNA-seq in two batches. At the same time, the endo-islets of recipient mice were also isolated and dissociated into single cells for scRNA-seq.

STZ-induced diabetic mice and islet transplantation were both performed as previously described [54]. Briefly, adult male and female C57BL/6J mice were selected as islet donors, and age-matched female C57BL/6J mice were chosen as the recipient. Female C57BL/6J mice were injected with STZ at the dose of 170 mg/kg after 6 h fasting. Mice that exhibited non-fasting hyperglycemia (>350 mg/kg) with 3 consecutive detections were regarded as diabetic mice for islet transplantation. Each diabetic mouse was transplanted with about 350 islets. About 1 month post-transplantation, the non-fasting blood glucose of diabetic mice recover normal (<144 mg/kg). These mice were selected for the oral glucose tolerance test.

### Preparation of single islet cells

Mouse islets were isolated from mice of different conditions, including 8-week-old healthy C57BL/6J mice of both sexes, 9-month-old healthy C57BL/6J mice of both sexes, 9-month-old T2D C57BL/6J mice of both sexes, and 11-month-old ICR mice of both sexes. For islet isolation, 0.5 mg/ml collagenase P (Catalog No. 11213873001, Roche, Basel, Switzerland) was poured into the pancreas by perfusion of the common bile duct. After digestion, islets were purified through Histopaque (Catalog Nos. 11191 and 10771, Sigma Aldrich, St. Louis, MO) gradient centrifugation. The Histopaque buffer was made by mixing Histopaque-10771 and Histopaque-11191 together at the ratio of 5:6. Purified islets were dissociated into single cells as follows: the islets were washed with cold PBS at least two times, spun at 1000 rpm for 2 min, and then the pellet was incubated with PBS containing 1 ml TrypLE Express (Catalog No. 12604021, Gibco, Grand Island, NY) at 37 °C for 10–15 min, with gentle cell pipetting using P1000 pipette. The reaction was stopped with low glucose DMEM (containing 10% FBS, 1% HEPES, and 1% PenStrep), and the cells were centrifuged at 1000 rpm for 2 min at 4 °C. Then the cell pellet was washed with cold PBS once, and the resuspended cells in PBS (containing 0.5% BSA) were filtered with a 40-micrometer strainer to generate single cell suspension. The transplanted islets were dissected from kidney capsule directly, and dissociated into single cells as described above. To obtain live single cell, Calcein Blue AM (Catalog No. C1429, Invitrogen, Carlsbad, CA) was used to measure the viability of cells, and single cells with high viability were sorted by using BD FACS Aria II flow cytometry (Pleasanton CA). Single islet cell was sorted into 96-well plates containing lysis buffer.

### Library construction and next-generation sequencing

scRNA-seq libraries were constructed according to the Smart-seq2 protocol except that oligo d(T) primers comprising 16-bp cell barcode sequence and 9-bp molecular barcode sequence were used to allow sample pooling and molecular counting. In addition, part of the TruSeq read2 sequence was used to replace the ISPCR sequence in the oligo d(T) primer thus to be compatible with Illumina sequence platform. Cells in lysis buffer were denatured, reverse transcribed in the presence of template switching oligos, and pre-amplified by adding both ISPCR and ISPCR-read2 primers with 24 PCR cycles. cDNA sequences of cells with different barcodes were then pooled together and purified using 0.8 × AMPure XP beads (Catalog No. A63881, Beckman, Brea, CA). Homemade Tn5 enzyme was used to tagment cDNA. Final amplification was processed using P7-index primers (TruSeq) and P5-index primers (Nextera). Sequencing libraries were purified with 0.6 × AMPure XP beads twice and with 1 × AMPure XP beads once, and then sequenced on Illumina HiSeq X10 platform with default parameters. Primer and adaptor sequences are all listed in Table S7.

### Quantitative real-time PCR

The cDNA for qRT-PCR assay was synthesized following the protocol that we described in the previous part (library construction). The β cells expressing *Ins2* from healthy and T2D mice of both sexes were identified by single cell qRT-PCR. qRT-PCR was performed with SuperReal PreMix Plus (SYBR Green) (Catalog No. EP205, TIANGEN, Beijing, China) by using the *Sdha* gene as an internal control. The relative expression levels of selected genes were analyzed based on the formula of 2^−ΔΔct^. The primers used for qRT-PCR are listed in Table S8.

### Physiology experiment

For intraperitoneal injection glucose tolerance test, ND and HFD feeding mice were fasted for 16 h. Then, glucose was intraperitoneally injected (1 g/kg body weight), and blood glucose was measured at time points of 0 min, 15 min, 30 min, 60 min, and 120 min after glucose injection using glucometer (Catalog No. ACCU-CHEK, Roche). Body weight was measured after 6 h fasting. For measurement of insulin level, blood samples were collected from mice that were fasted for 6 h, and the insulin level was measured by using Mouse Ultrasensitive Insulin ELISA Kit (Catalog No. 80-INSMSU-E01, ALPOC, Salem, NH). For oral glucose tolerance test, mice were fasted for 6 h, and glucose was gavaged at a dose of 2 g/kg body weight. Tail blood was collected for blood glucose detection, at time points of 0 min, 15 min, 30 min, 60 min, and 120 min, using glucometer (Catalog No. ACCU-CHEK, Roche).

### Quantification and statistical analysis

#### Preprocessing before normalization

Reads were stored in paired-end fastq format. Reads of one end of a fragment containing cell barcode/UMI information were subsequently extracted and added to the name of corresponding reads of the other end in fastq file containing molecular sequence. That barcode/UMI information was in fastq-R2 files. Next, the generated single-end fastq files were cleaned by Trim Galore (http://www.bioinformatics.babraham.ac.uk/projects/trim_galore/) with the parameter—length 30.

Then, FastQC (https://www.bioinformatics.babraham.ac.uk/projects/fastqc/) was applied to check read quality followed by alignment on mm9 genome using STAR [55]. The parameters of STAR software were set as following: “Align Ends Type End To End”, “out FileterMismatchNoverReadLmax = 0.04”, “out SAMattrIHstart = 0”, “out SAMmultNmax = 1”, and “out FilterMultimapNmax = 1”. GTF file of mm9 reference genome was derived from the RefSeq gene annotation [56] file downloaded from UCSC genome browser database (http://genome.ucsc.edu/cgi-bin/hgGateway?db=mm9). After alignment, SAM files underwent demultiplexing by Catadapt (https://github.com/marcelm/cutadapt/releases) with parameters “overlap 16-bp, no-indels, match-read-wildcards”; reads were removed if they were assigned to more than one cell. Then, we removed PCR duplicates by UMI-tools [57]. Next, feature counts [58] were utilized to quantify gene expression levels according to the aforementioned GTF file. Once the expression profiles were generated, cells with fewer than 500 expressed genes (UMI count > 1) were considered low-quality and were removed (Figure S1). The fraction of transcripts from mitochondrial genes in each single cell was investigated as it was used as an indicator for general quality control. Part of our ICR cells had high transcript fraction of mitochondrial genes, which can be accounted for that kidney cells intrinsically express high-level mitochondrial genes [59]. So, we kept those cells for analysis because our transplantation cells were under renal capsules and either kidney cells or affected transplanted cells may well be introduced in our data. Next, only genes expressed (UMI count > 1) in 5 or more cells were used for further analysis. Eventually, there are 14,152 genes and 4662 cells retained in mouse scRNA-seq data. The sample information of the Illumina high-throughput sequencing data is listed in Table 1.

#### Normalization

Here we used an adjCPM normalization method. The CPM method assumes that the total reads are equal among cells. Similarly, our adjCPM method has an assumption except excluding a few genes in calculating the total number of reads of each cell. It is based on the observation that sometimes a few top expressed genes can account for more than 50% total UMI count (Figure S1). These genes were selected as union set of the top 2 expressed genes of single cells, in which the sum of UMI counts for the corresponding top 2 genes is beyond 50% of the total UMI count of the cell. Specifically, we obtained 9 genes (*Gcg*, *Ins1*, *Ins2*, *Malat1*, *Ppy*, *Pyy*, *Rn45s*, *Ttr*, and *mt-Rnr2*) and they were excluded when calculating the total UMI count of each cell. It is well known that the scRNA-seq experiments are affected by dropout events especially for the UMI method. Here, we also observed that many genes have zero expression value because of insufficient detection power or intrinsically under-expression of these genes. To address this issue, SAVER [60] was applied to recover gene expression in the entire matrix.

### Cell type identification

Since cell type identification is critical for the following analyses, we took two steps to define the cell types robustly. Firstly, we selected the top 10 expressed genes of each cell, leading to a total of 118 genes to perform the subsequent hieratical clustering analysis. Accordingly, we primarily assigned 4 clusters of single cells after removing 111 cells that had ulterior distance in clustering dendrogram, and annotated each cluster based on the expression of marker genes (Figure S1F).

Secondly, by a lowess regression between the log_2_ mean and log_2_ coefficient of variance of normalized UMI count for each gene across all single cells, we selected 3000 genes according to the residue of lowess regression as HVGs. Next, PCA was applied to the log_2_-transformed normalized expression of these HVGs using *sklearn* [61] after centering and scaling, and top 25 PCs were selected based on the statistical significance of the fraction of total variance explained by each of them, which was estimated using jackstraw [62]. We used the top 25 PCs as input for subsequent t-SNE analysis. To have a stable t-SNE visualization, we tried a lot of combinations of parameters [perplexing (10, 15, 20, 25, 30], early exaggeration (12, 15, 20), learning rate (200, 300, 400, 500, 600, 800), and 50 random seeds] using *sklearn* [62], and found that they generally gave very similar results. We finally chose the following settings—perplexing (15), early exaggeration (12), and learning rate (500)—to generate the t-SNE plots shown in the figures. Next, Density-Based Spatial Clustering of Applications with Noise (DBSCAN) algorithm, [63] with parameters “eps = 5 and MinPts = 5” was applied on the resulting two-dimensional t-SNE map to group the single cells into cliques (Figure S1G). After removing singletons, we examined each single cell clique in order of clique size with following criteria: 1) only cells with consistent cluster/clique labels generated by hierarchical clustering using the top 10 expressed genes and by DBSCAN were retained; 2) differential expression analysis of single cells in clique 3 (C3) compared with the single cells in other cliques demonstrated that the single cells in C3 should be excluded because they highly expressed cell cycle-related genes (Figure S1H). Finally, we kept 4467 high-quality mouse single cells for downstream analysis, and the cell type labels were inferred from the marker genes highly expressed in each single cell clique (Figure S1I).

### Differential expression analysis and GSEA

MAST [17] was used to perform differential expression analysis of scRNA-seq profiles of β cells between male and female or between T2D and healthy mice. CDR, which means the number of genes detected in each single cell, was controlled as a covariate while estimating treatment effects. Significantly regulated genes were identified by using FDR < 0.05 as cutoff. For identification of functional pathways enriched in the detected DEGs between different sexes or between T2D and healthy mice in our scRNA-seq data, we performed GSEA [19] on genes ranked based on their z-statistics, which were derived by mapping their MAST *P* values to the standard normal distribution and using the sign of the log_2_ FC of their expression values to represent the direction of regulation. Technically, the z-statistic of each gene was calculated as:

qnorm(p) × sign(lfc)

where p and lfc are *P* value and log_2_ FC of this gene obtained from MAST output, respectively; qnorm is the standard normal quantile function and sign is the signum function.

At the same time, we also tried other methods to perform differential expression analysis, such as edgeR. Performing differential analysis using edgeR combined with zingeR to incorporate an estimate of the dropout rate per cell (edgeR-zingeR), which is a DE-tool for UMI-based scRNA-seq data [64]. Using the same cutoff of MAST, abundant DEGs were also identified by edgeR-zingeR. In general, most significant DEGs from two separate methods are similar, most of which detected by MAST could also be covered by edgeR-zingeR (Figure S4), suggesting that the results of MAST are stable and could be repeated by another differential tool. The DEGs detected by edgeR are listed in Table S9 for reference. KEGG pathways [65] were used as input gene sets for GSEA, and FDR cutoff of < 0.25 was used to select statistically significant pathways.

## Ethical statement

All experiments were performed in accordance with the University of Health Guide for the Care and Use of Laboratory Animals and were approved by the Biological Research Ethics Committee of Tongji University.

## Data availability

Raw data (Fastq files) for single pancreatic β cell RNA-seq in this study have been submitted to Genome Sequence Archive [66] at the National Genomics Data Center, Beijing Institute of Genomics, Chinese Academy of Sciences / China National Center for Bioinformation (GSA: CRA003921), and are publicly accessible at https://ngdc.cncb.ac.cn/gsa.

## Competing interests

The authors have declared no competing interests.

### CRediT authorship contribution statement

**Gang Liu:** Conceptualization, Methodology, Validation, Investigation, Visualization, Writing – original draft, Writing – review & editing. **Yana Li:** Software, Formal analysis, Data curation, Visualization, Data curation, Writing – original draft, Writing – review & editing. **Tengjiao Zhang:** Methodology, Resources, Investigation, Writing – original draft. **Mushan Li:** Software, Formal analysis, Data curation, Writing – original draft. **Sheng Li:** Validation, Resources. **Qing He:** Validation, Resources. **Shuxin Liu:** Resources. **Minglu Xu:** Resources. **Tinghui Xiao:** Resources. **Zhen Shao:** Visualization, Supervision, Writing – original draft, Writing – review & editing, Project administration, Funding acquisition. **Weiyang Shi:** Visualization, Supervision, Writing – original draft, Project administration, Funding acquisition. **Weida Li:** Conceptualization, Visualization, Supervision, Project administration, Funding acquisition.

## Figures and Tables

**Supplementary figure S32:**
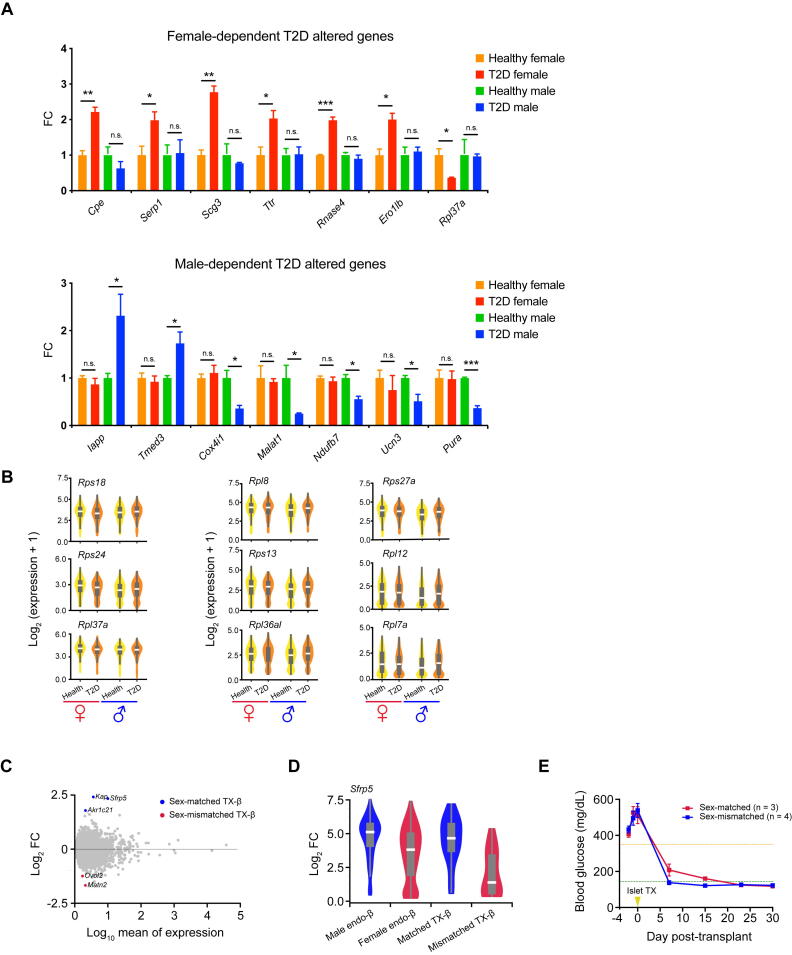


**Supplementary figure S4:**