# Correction to: Single-cell RNA Sequencing Reveals Sexually Dimorphic Transcriptome and Type 2 Diabetes Genes in Mouse Islet β Cells

**DOI:** 10.1093/gpbjnl/qzae022

**Published:** 2024-07-09

This is a correction to: Gang Liu, Yana Li, Tengjiao Zhang, Mushan Li, Sheng Li, Qing He, Shuxin Liu, Minglu Xu, Tinghui Xiao, Zhen Shao, Weiyang Shi, Weida Li, Single-cell RNA Sequencing Reveals Sexually Dimorphic Transcriptome and Type 2 Diabetes Genes in Mouse Islet β Cells, *Genomics, Proteomics & Bioinformatics*, Volume 19, Issue 3, June 2021, Pages 408–422, https://doi.org/10.1016/j.gpb.2021.07.004.

The editors regret that there were errors in Figures 2F, 3E, and 4D published in Issue 3, 2021. In Figures 2F, 3E, and 4D, the colors of the heatmaps did not match their corresponding color scales. These errors were caused during the production process by former publisher. The corrected Figures 2, 3, and 4 are shown below. The editors would like to apologize for any inconvenience caused.

**Figure 2:**
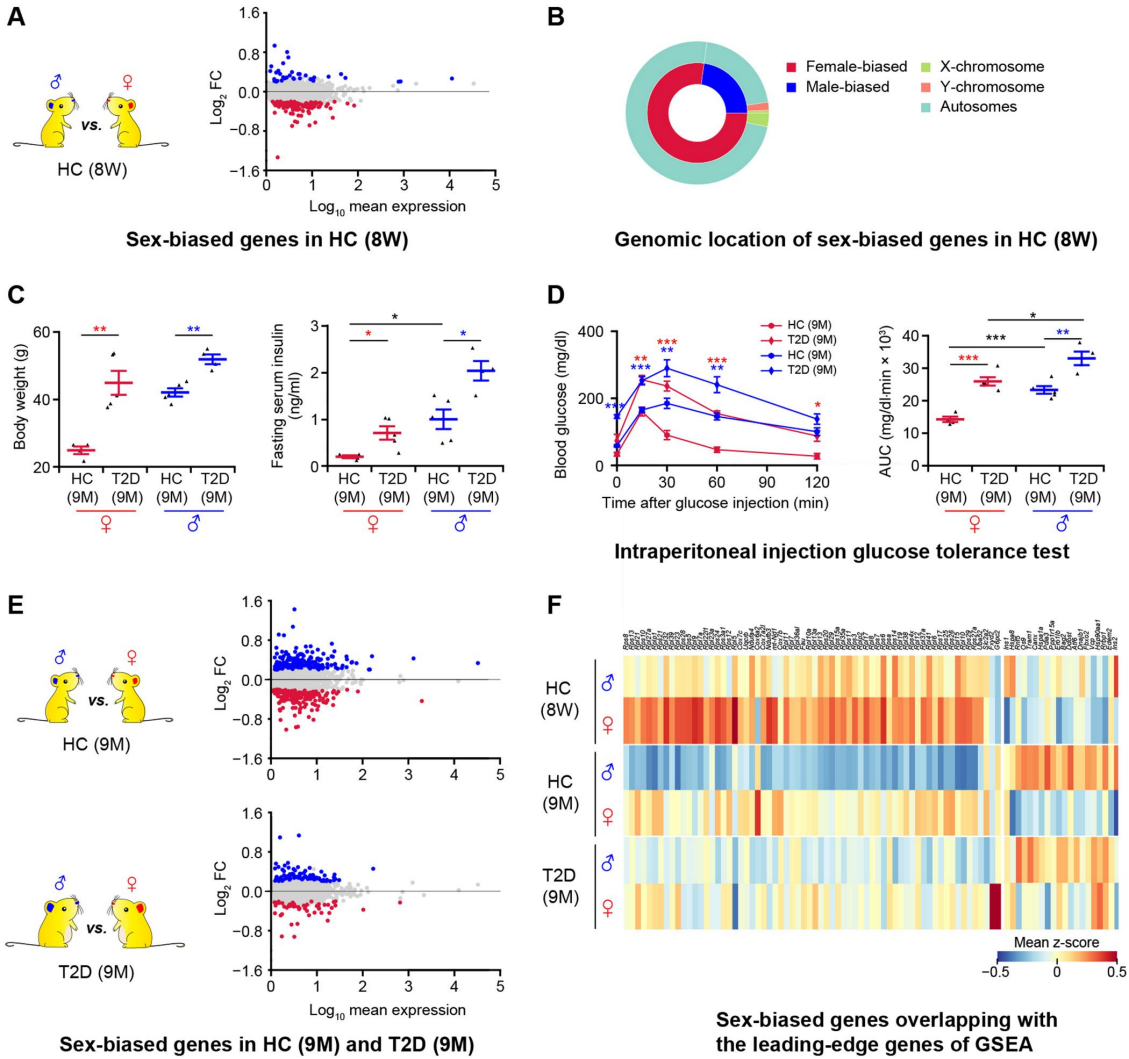
Sex-biased genes exist in both healthy and T2D mice**A**. Differential gene expression analysis between β cells in 8-week-old male and female HC C57BL/6J mice. In the MA plot, the male-biased and female-biased genes are indicated in blue and red, respectively. Gene expression levels are calculated as log_2_ normalized UMI counts and the obtained values are further log_10_ transformed for easy view in the plot. More details are provided in Table S1. **B**. Nested pie chart depicting the genomic location of sex-biased genes. **C**. Diabetes-associated physiological phenotypes. Body weight and serum insulin level are detected after 6 h fasting. The blue dots represent the male HC (*n* = 5) and T2D (*n* = 4) mice; the red dots represent the female HC (*n* = 4) and T2D (*n* = 5) mice. Data are presented as mean ± SEM. *, *P* < 0.05; **, *P* < 0.01 (two-sample *t*-test). **D**. Intraperitoneal injection glucose tolerance test. Time course is shown on the left and AUC values are shown on the right. The blue lines represent the male HC (*n* = 5) and T2D (*n* = 4) mice; the red lines represent the female HC (*n* = 4) and T2D (*n* = 5) mice. Red asterisks represent female T2D *vs*. female HC mice; blue asterisks represent male T2D *vs*. male HC mice. Data are presented as mean ± SEM. *, *P* < 0.05; **, *P* < 0.01; ***, *P* < 0.001 (two-sample *t*-test). **E**. Differential gene expression analysis between β cells in 9-month-old male and female HC and T2D mice. In the MA plots, the male-biased and female-biased genes are indicated in blue and red, respectively. More details are provided in Tables S2 and S3. **F**. Heatmap showing sex-biased genes overlapping with the leading-edge genes of GSEA. The color scale shows the mean value of z-score, with blue and red corresponding to the minimum and maximum values of standardized log_2_ (expression + 1), respectively. More details are provided in Table S4. DEG, differentially expressed gene; GSEA, gene set enrichment analysis; AUC, area under curve.

**Figure 3:**
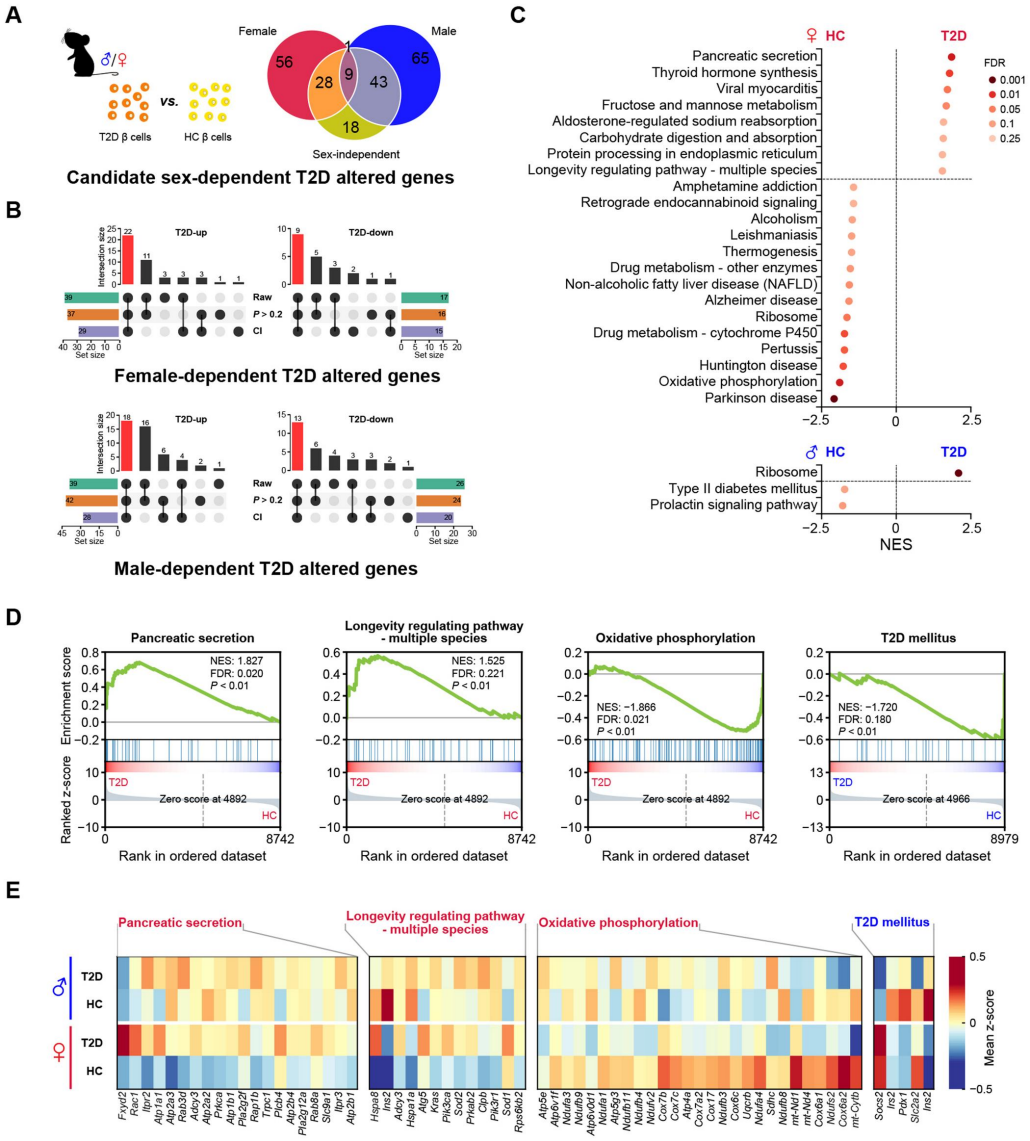
T2D altered genes and pathways differ in a sex-dependent manner**A**. Venn diagram depicting the definition of potential sex-dependent T2D altered genes. **B**. Upset plot shows the definition of sex-dependent T2D altered genes. The red bar shows the number of sex-dependent T2D altered genes in each group. “Raw” represents the candidate genes defined in Figure 3A. “CI” represents the genes whose CI of log_2_ FC from analysis of one sex does not overlap with that from the opposite sex. “*P* > 0.2” represents that the *P* values in the non-significant sex group are high. The bar chart at bottom left or right represents the number of genes under each filter condition. The bar chart above the dot plot represents the number of genes in each group that fit different filter condition (black dot). More details are provided in Table S6. **C**. Results of GSEA showing pathways significantly enriched in the HC group and the T2D group in female (labeled in red) and male (labeled in blue) mice. Pathways with NES > 0 are enriched in β cells of T2D mice, and pathways with NES < 0 are enriched in β cells of HC mice. **D**. GSEA plots of pathways involved in the onset of T2D or the function of β cells. **E**. Heatmap showing leading-edge genes of GSEA included in the selected pathways. The pathway in red represents the female-specific enriched pathway in β cells of T2D or HC mice. The pathway in blue represents male-specific enriched pathway in β cells of HC mice. T2D-up, T2D up-regulated gene; T2D-down, T2D down-regulated gene; CI, confidence interval; NES, normalized enrichment score.

**Figure 4:**
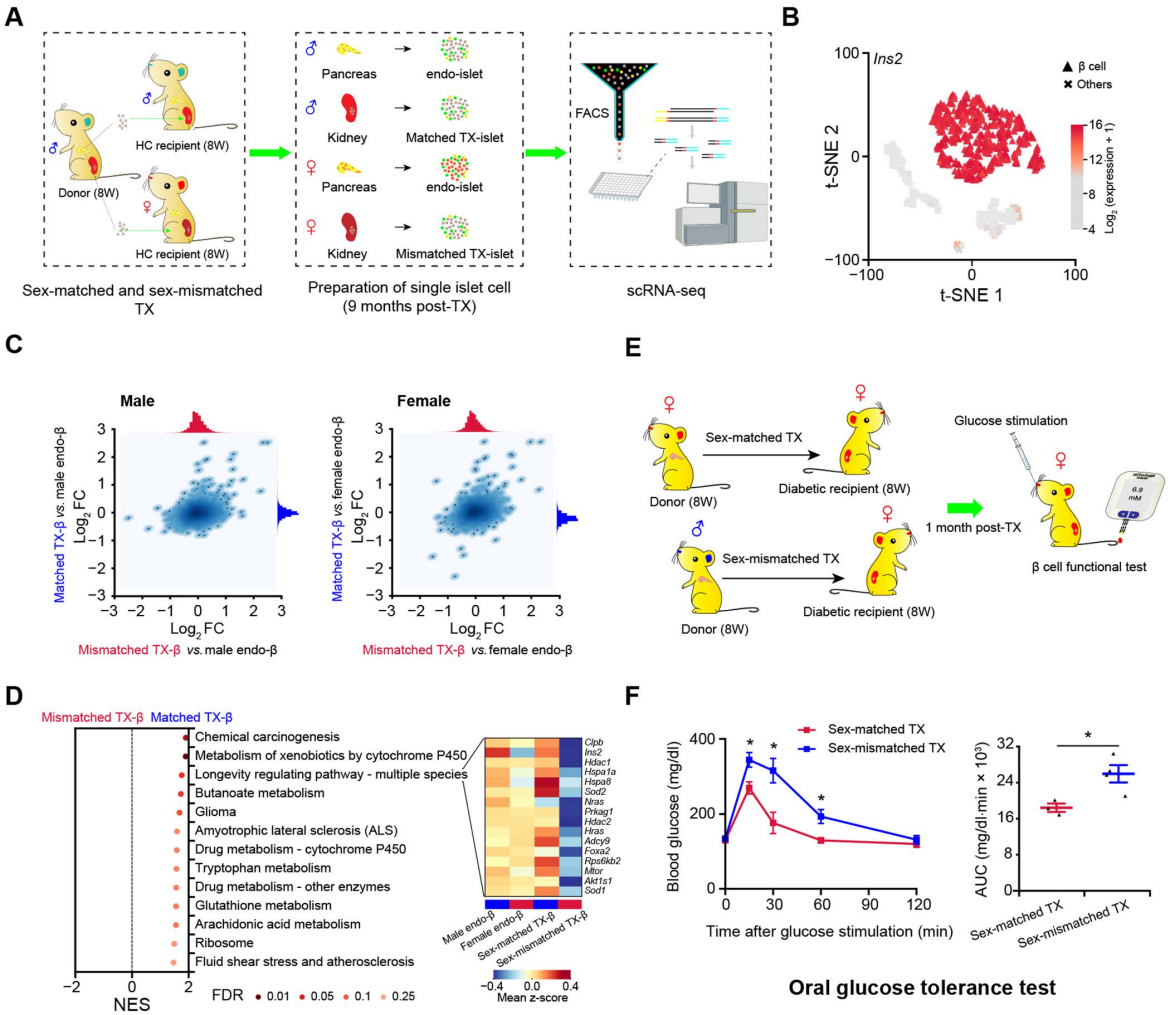
Sex-matched islet TX confers better glucose tolerance than sex-mismatched transplantation**A**. Schematic diagram of sex-matched and sex-mismatched islet TX for scRNA-seq. Single islet cells are collected for scRNA-seq 9 months post-TX. **B**. t-SNE with cell type information. β cells from endo-islet and TX-islet cells in both sex-matched and sex-mismatched TX experiments are identified by high expression of *Ins2*. More details are provided in Figure S2A. **C**. Correlation analysis between TX-β and endo-β cells. Scatterplot depicting correlation of log_2_ FC values generated by comparing TX-β cells (sex-matched or sex-mismatched) to male endo-β cells (left panel) or female endo-β cells (right panel). **D**. GSEA of sex-matched and sex-mismatched TX-β cells. Leading-edge genes of the longevity regulating pathway are zoomed in with heatmap. **E**. Schematic diagram of sex-matched and sex-mismatched islet TX for β cell functional test in STZ-induced female diabetic mice. **F**. Oral glucose tolerance test for mice after sex-matched and sex-mismatched islet TX. Time course is shown on the left and AUC values are shown on the right. Glucose (2 g/kg body weight) is gavaged after 6 h fasting, and blood glucose level is detected at 0 min, 15 min, 30 min, 60 min, and 120 min after glucose gavage. The blue line represents the sex-mismatched islet TX (*n* = 4); the red line represents sex-matched islet TX (*n* = 3). Data are presented as mean ± SEM. *, *P* < 0.05 (two-sample *t*-test). TX, transplanted/transplantation; endo-islet, endogenous islet of recipient mouse; endo-β, endogenous β cell of recipient mouse; TX-β, transplanted β cell; STZ, streptozotocin.

## Supplementary Material

qzae022_Supplementary_Data

