# Supplementary material for: Single-cell RNA Sequencing Reveals Sexually Dimorphic Transcriptome and Type 2 Diabetes Genes in Mouse Islet β Cells
Source: Genomics Proteomics Bioinformatics. 2021 Sep 24;19(3):408–22. doi: 10.1016/j.gpb.2021.07.004 (PMC8864195; doi:10.1016/j.gpb.2021.07.004)
Supplement: Supplementary Table S3 [file mmc4.docx]

**Table S3 Sex-biased genes in β cells of 9-month-old T2D mice**

| **Type** | **Gene symbol** | **BaseMean** | **Log_2_ fold change** | | ***P* value** | ***P* adjust** |
| --- | --- | --- | --- | --- | --- | --- |
| Male-biased genes | *Eif2s3y* | 1.565417 | 1.094034 | 1.91E–81 | | 8.76E–78 |
|  | *Scg2* | 169.1795 | 0.457586 | 1.63E–48 | | 4.97E–45 |
|  | *Ddx3y* | 1.276198 | 0.562089 | 9.94E–44 | | 1.83E–40 |
|  | *Necab2* | 4.042517 | 1.133534 | 8.11E–38 | | 1.24E–34 |
|  | *Uty* | 1.219289 | 0.463558 | 4.86E–37 | | 6.38E–34 |
|  | *Naa20* | 1.770286 | 0.439966 | 1.36E–15 | | 8.93E–13 |
|  | *Rnf5* | 1.982972 | 0.523402 | 2.64E–15 | | 1.62E–12 |
|  | *Nkx2–2* | 2.410468 | 0.479397 | 1.24E–13 | | 6.69E–11 |
|  | *Sdf2l1* | 3.862331 | 0.489877 | 1.13E–12 | | 5.79E–10 |
|  | *Gc* | 2.332014 | 0.577055 | 1.39E–12 | | 6.71E–10 |
|  | *Tmed2* | 2.888774 | 0.356069 | 3.74E–12 | | 1.72E–09 |
|  | *Tssc4* | 6.950201 | 0.47455 | 4.75E–11 | | 1.98E–08 |
|  | *Spp1* | 1.449726 | 0.494258 | 2.80E–10 | | 1.12E–07 |
|  | *P4hb* | 14.77533 | 0.321963 | 5.18E–10 | | 1.90E–07 |
|  | *Bambi* | 1.91103 | 0.409253 | 1.55E–09 | | 5.26E–07 |
|  | *Cd47* | 3.328244 | 0.423413 | 9.48E–09 | | 2.81E–06 |
|  | *Syp* | 5.164722 | 0.370449 | 1.47E–08 | | 4.10E–06 |
|  | *Il1r1* | 12.50519 | 0.398575 | 1.89E–08 | | 5.10E–06 |
|  | *Mrps28* | 1.231113 | 0.213988 | 2.95E–08 | | 7.74E–06 |
|  | *Rasgrf2* | 1.315226 | 0.280902 | 7.52E–08 | | 1.87E–05 |
|  | *Paip2* | 8.160833 | 0.330627 | 1.26E–07 | | 2.97E–05 |
|  | *Atn1* | 1.282771 | 0.241061 | 2.58E–07 | | 5.64E–05 |
|  | *Hyou1* | 3.048203 | 0.346366 | 2.88E–07 | | 6.14E–05 |
|  | *Pappa2* | 1.325729 | 0.292922 | 7.20E–07 | | 1.44E–04 |
|  | *Meg3* | 23.47778 | 0.284943 | 1.00E–06 | | 1.95E–04 |
|  | *Rnase4* | 24.25724 | 0.339201 | 1.20E–06 | | 2.26E–04 |
|  | *Ccnd1* | 2.574984 | 0.346763 | 1.49E–06 | | 2.54E–04 |
|  | *Kmt2d* | 4.413322 | 0.354876 | 1.61E–06 | | 2.68E–04 |
|  | *Acly* | 11.33837 | 0.247448 | 1.97E–06 | | 3.17E–04 |
|  | *Tspan33* | 5.578722 | 0.414638 | 2.61E–06 | | 4.13E–04 |
|  | *Pdia3* | 30.58145 | 0.227263 | 3.78E–06 | | 5.64E–04 |
|  | *Tmbim6* | 5.873785 | 0.287399 | 3.81E–06 | | 5.64E–04 |
|  | *Kctd13* | 1.484975 | 0.249898 | 5.14E–06 | | 7.48E–04 |
|  | *Nnt* | 1.49006 | 0.24099 | 5.76E–06 | | 8.27E–04 |
|  | *Tram1* | 5.606432 | 0.30353 | 5.89E–06 | | 8.31E–04 |
|  | *Hap1* | 4.261206 | 0.358741 | 6.83E–06 | | 9.43E–04 |
|  | *Slc16a10* | 3.129277 | 0.325063 | 6.88E–06 | | 9.43E–04 |
|  | *Dtx3* | 2.307832 | 0.308141 | 7.25E–06 | | 9.79E–04 |
|  | *Derl3* | 1.37897 | 0.230422 | 1.21E–05 | | 1.48E–03 |
|  | *Selenof* | 9.825785 | 0.276931 | 1.28E–05 | | 1.55E–03 |

**Table S3 Sex-biased genes in β cells of 9-month-old T2D mice (continued)**

| **Type** | **Gene symbol** | **BaseMean** | **Log_2_ fold change** | ***P* value** | ***P* adjust** |
| --- | --- | --- | --- | --- | --- |
| Male-biased genes | *Dapl1* | 3.094251 | 0.451124 | 1.44E–05 | 1.72E–03 |
|  | *Pdia4* | 7.161075 | 0.326121 | 2.07E–05 | 2.40E–03 |
|  | *Tent5a* | 1.646579 | 0.285574 | 2.34E–05 | 2.65E–03 |
|  | *Itm2c* | 3.896665 | 0.275278 | 3.73E–05 | 4.17E–03 |
|  | *Copb2* | 6.644442 | 0.273293 | 3.83E–05 | 4.24E–03 |
|  | *Manf* | 25.23628 | 0.249507 | 3.95E–05 | 4.32E–03 |
|  | *Syt13* | 23.49679 | 0.225319 | 4.45E–05 | 4.75E–03 |
|  | *Clic4* | 1.596611 | 0.229493 | 5.16E–05 | 5.45E–03 |
|  | *Cst3* | 11.12087 | 0.266013 | 7.00E–05 | 7.06E–03 |
|  | *Pcp4* | 1.916138 | 0.327611 | 8.85E–05 | 8.73E–03 |
|  | *Etfb* | 7.315348 | 0.257408 | 1.06E–04 | 1.02E–02 |
|  | *Gadd45gip1* | 6.820377 | 0.275969 | 1.08E–04 | 1.02E–02 |
|  | *Sdhd* | 2.0884 | 0.275772 | 1.13E–04 | 1.05E–02 |
|  | *Akap8l* | 2.601281 | 0.315681 | 1.43E–04 | 1.30E–02 |
|  | *Rab3gap2* | 1.472903 | 0.208792 | 1.62E–04 | 1.42E–02 |
|  | *Unc50* | 1.75063 | 0.212947 | 1.78E–04 | 1.53E–02 |
|  | *Ptprn* | 8.325304 | 0.312999 | 2.00E–04 | 1.70E–02 |
|  | *Vegfa* | 1.752899 | 0.227135 | 2.04E–04 | 1.70E–02 |
|  | *Kif12* | 5.181117 | 0.278108 | 2.16E–04 | 1.79E–02 |
|  | *Sdc4* | 1.809251 | 0.228347 | 2.39E–04 | 1.94E–02 |
|  | *Spcs3* | 6.360416 | 0.249628 | 2.45E–04 | 1.97E–02 |
|  | *Cyb561* | 3.192056 | 0.284948 | 3.78E–04 | 2.80E–02 |
|  | *Rab34* | 2.469559 | 0.252766 | 4.01E–04 | 2.92E–02 |
|  | *Fxyd6* | 4.160209 | 0.296018 | 4.24E–04 | 3.02E–02 |
|  | *Ldlr* | 2.613563 | 0.254162 | 4.36E–04 | 3.06E–02 |
|  | *Spint2* | 8.66128 | 0.240165 | 4.39E–04 | 3.06E–02 |
|  | *Dio1* | 3.979043 | 0.300937 | 4.41E–04 | 3.06E–02 |
|  | *Chic1* | 16.12917 | 0.206386 | 4.46E–04 | 3.08E–02 |
|  | *Nbas* | 4.752153 | 0.241714 | 4.74E–04 | 3.19E–02 |
|  | *Sdhc* | 3.191769 | 0.273008 | 4.85E–04 | 3.23E–02 |
|  | *Rsrp1* | 12.64166 | 0.211453 | 5.33E–04 | 3.47E–02 |
|  | *1700086L19Rik* | 1.540502 | 0.228366 | 5.39E–04 | 3.48E–02 |
|  | *Dnajc3* | 19.45073 | 0.205506 | 5.47E–04 | 3.50E–02 |
|  | *Selenom* | 9.347638 | 0.249914 | 5.56E–04 | 3.50E–02 |
|  | *H1f0* | 3.587263 | 0.291541 | 5.58E–04 | 3.50E–02 |
|  | *2900055J20Rik* | 2.526588 | 0.235727 | 5.60E–04 | 3.50E–02 |
|  | *Sec11c* | 16.56363 | 0.232702 | 5.77E–04 | 3.55E–02 |
|  | *Abcc8* | 13.96821 | 0.20816 | 6.05E–04 | 3.70E–02 |
|  | *Hid1* | 4.301314 | 0.268494 | 6.16E–04 | 3.75E–02 |
|  | *Csad* | 2.03415 | 0.255615 | 6.81E–04 | 4.06E–02 |
|  | *Rpn1* | 13.91703 | 0.23034 | 7.13E–04 | 4.17E–02 |

**Table S3 Sex-biased genes in β cells of 9-month-old T2D mice (continued)**

| **Type** | **Gene symbol** | **BaseMean** | **Log_2_ fold change** | ***P* value** | ***P* adjust** |
| --- | --- | --- | --- | --- | --- |
| Female-biased genes | *Xist* | 2.153581 | –1.93706 | 2.46E–160 | 2.26E–156 |
|  | *Cish* | 1.716479 | –0.91719 | 1.66E–46 | 3.81E–43 |
|  | *Enpp2* | 3.210527 | –0.92367 | 2.43E–36 | 2.79E–33 |
|  | *Socs2* | 1.616638 | –0.49001 | 4.53E–21 | 4.62E–18 |
|  | *G6pc2* | 102.8299 | –0.37441 | 1.09E–19 | 1.00E–16 |
|  | *Prlr* | 17.2077 | –0.59562 | 1.61E–19 | 1.34E–16 |
|  | *Jup* | 3.764578 | –0.66213 | 7.84E–19 | 6.00E–16 |
|  | *Fxyd2* | 1.214431 | –0.44606 | 4.47E–16 | 3.15E–13 |
|  | *Gcg* | 2.79143 | –0.67637 | 3.57E–15 | 2.05E–12 |
|  | *Cebpd* | 1.255522 | –0.28866 | 8.25E–10 | 2.91E–07 |
|  | *Prss53* | 21.08724 | –0.33773 | 2.60E–09 | 8.52E–07 |
|  | *Sytl4* | 6.728527 | –0.48697 | 3.96E–09 | 1.25E–06 |
|  | *Chga* | 652.1322 | –0.22814 | 8.70E–09 | 2.66E–06 |
|  | *Rpl23* | 18.66599 | –0.44043 | 1.39E–08 | 4.00E–06 |
|  | *Spc25* | 12.58776 | –0.43854 | 4.88E–08 | 1.24E–05 |
|  | *Cd9* | 1.187818 | –0.22565 | 9.17E–08 | 2.21E–05 |
|  | *Rpl10* | 17.59557 | –0.27475 | 2.09E–07 | 4.80E–05 |
|  | *Sphkap* | 8.868342 | –0.43345 | 2.30E–07 | 5.14E–05 |
|  | *Gpx3* | 1.732163 | –0.46512 | 6.61E–07 | 1.35E–04 |
|  | *Galnt9* | 1.266092 | –0.22996 | 1.24E–06 | 2.27E–04 |
|  | *Chst12* | 1.480606 | –0.23741 | 1.33E–06 | 2.39E–04 |
|  | *Hsp90ab1* | 25.31112 | –0.28855 | 1.48E–06 | 2.54E–04 |
|  | *P2ry1* | 1.934401 | –0.31061 | 1.84E–06 | 3.02E–04 |
|  | *Rps14* | 23.28303 | –0.21766 | 3.21E–06 | 4.92E–04 |
|  | *Fmo1* | 1.289827 | –0.24252 | 7.68E–06 | 1.02E–03 |
|  | *Hsbp1* | 5.470287 | –0.34831 | 8.27E–06 | 1.06E–03 |
|  | *Rplp1* | 10.92367 | –0.323 | 8.28E–06 | 1.06E–03 |
|  | *Hnrnpm* | 4.080875 | –0.36314 | 8.39E–06 | 1.06E–03 |
|  | *Ap1s2* | 4.147844 | –0.3482 | 8.46E–06 | 1.06E–03 |
|  | *Akr1c19* | 2.210838 | –0.3333 | 5.91E–05 | 6.17E–03 |
|  | *Fau* | 9.932548 | –0.29834 | 6.35E–05 | 6.55E–03 |
|  | *Cox7a2l* | 6.08677 | –0.27174 | 7.75E–05 | 7.73E–03 |
|  | *Vps35* | 5.180932 | –0.29223 | 1.02E–04 | 9.94E–03 |
|  | *Rpl21* | 14.69273 | –0.27514 | 1.48E–04 | 1.33E–02 |
|  | *Peg3* | 3.095583 | –0.30852 | 1.58E–04 | 1.40E–02 |
|  | *Gcsh* | 1.46553 | –0.20959 | 1.61E–04 | 1.42E–02 |
|  | *Rlf* | 1.758805 | –0.24176 | 2.02E–04 | 1.70E–02 |
|  | *Stip1* | 2.542513 | –0.26871 | 2.58E–04 | 2.04E–02 |
|  | *Gadd45g* | 3.342915 | –0.30432 | 3.25E–04 | 2.52E–02 |
|  | *Rpl27a* | 2.966972 | –0.22878 | 3.26E–04 | 2.52E–02 |

**Table S3 Sex-biased genes in β cells of 9-month-old T2D mice (continued)**

| **Type** | **Gene symbol** | **BaseMean** | **Log_2_ fold change** | ***P* value** | ***P* adjust** |
| --- | --- | --- | --- | --- | --- |
| Female-biased genes | *Mt1* | 11.11573 | –0.35406 | 3.49E–04 | 2.63E–02 |
|  | *Imp3* | 2.583336 | –0.30017 | 3.95E–04 | 2.90E–02 |
|  | *Hist1h2bc* | 5.597822 | –0.30283 | 4.04E–04 | 2.92E–02 |
|  | *Rpl13a* | 25.5201 | –0.20481 | 4.19E–04 | 3.01E–02 |
|  | *Rgs2* | 6.067359 | –0.39791 | 4.55E–04 | 3.12E–02 |
|  | *Echdc2* | 2.415706 | –0.29484 | 4.70E–04 | 3.19E–02 |
|  | *Eif3m* | 3.160661 | –0.25902 | 4.91E–04 | 3.24E–02 |
|  | *Slc2a2* | 11.02216 | –0.26252 | 5.54E–04 | 3.50E–02 |
|  | *Zbtb20* | 3.904341 | –0.32015 | 6.85E–04 | 4.06E–02 |
|  | *Uba52* | 7.592675 | –0.20725 | 7.02E–04 | 4.13E–02 |
|  | *Psma6* | 3.12481 | –0.26667 | 7.22E–04 | 4.20E–02 |
|  | *2210016F16Rik* | 2.860618 | –0.27711 | 7.88E–04 | 4.47E–02 |
