# Supplementary material for: Single-cell RNA Sequencing Reveals Sexually Dimorphic Transcriptome and Type 2 Diabetes Genes in Mouse Islet β Cells
Source: Genomics Proteomics Bioinformatics. 2021 Sep 24;19(3):408–22. doi: 10.1016/j.gpb.2021.07.004 (PMC8864195; doi:10.1016/j.gpb.2021.07.004)
Supplement: Supplementary Table S5 [file mmc6.docx]

**Table S5 Sex-independent T2D altered genes**

| **Type** | **Gene symbol** | **BaseMean** | **Log_2_ fold change** | ***P* value** | ***P* adjust** |
| --- | --- | --- | --- | --- | --- |
| Up-regulated genes | *Rgs2* | 6.981633 | 0.780859 | 2.97E–27 | 2.70E–23 |
|  | *Dapl1* | 2.589408 | 0.524064 | 2.17E–15 | 3.95E–12 |
|  | *Gcg* | 3.760926 | 0.409930 | 1.17E–12 | 1.33E–09 |
|  | *Fh1* | 2.952059 | 0.356861 | 4.55E–12 | 4.14E–09 |
|  | *Dnajb9* | 7.010756 | 0.323539 | 9.40E–12 | 7.13E–09 |
|  | *Pde1c* | 1.498825 | 0.221218 | 6.76E–11 | 4.39E–08 |
|  | *Rap1gap2* | 4.492751 | 0.339940 | 5.76E–09 | 2.28E–06 |
|  | *Gng4* | 3.350993 | 0.295577 | 9.17E–09 | 3.46E–06 |
|  | *Tmem39a* | 2.260753 | 0.253299 | 1.15E–08 | 4.02E–06 |
|  | *Pcbp1* | 4.711200 | 0.344865 | 1.28E–08 | 4.31E–06 |
|  | *Degs1* | 11.015235 | 0.286595 | 1.42E–08 | 4.62E–06 |
|  | *Pabpc1* | 12.942235 | 0.354198 | 2.62E–08 | 8.23E–06 |
|  | *Wnt4* | 3.749900 | 0.333265 | 4.68E–08 | 1.37E–05 |
|  | *Creld2* | 8.807488 | 0.341376 | 5.19E–08 | 1.43E–05 |
|  | *Hsp90aa1* | 13.160620 | 0.298023 | 7.34E–08 | 1.96E–05 |
|  | *Hars* | 2.194724 | 0.270855 | 8.50E–08 | 2.21E–05 |
|  | *Bhlha15* | 1.958745 | 0.248461 | 1.24E–07 | 3.05E–05 |
|  | *Dpagt1* | 1.627566 | 0.212566 | 1.67E–07 | 3.89E–05 |
|  | *Ubr4* | 4.973512 | 0.308925 | 2.08E–07 | 4.73E–05 |
|  | *H2afy* | 3.785894 | 0.307656 | 3.01E–07 | 6.51E–05 |
|  | *Ccnd1* | 2.207646 | 0.253625 | 3.43E–07 | 7.25E–05 |
|  | *Srm* | 1.731855 | 0.204229 | 4.77E–07 | 9.65E–05 |
|  | *Pcp4* | 1.671257 | 0.271585 | 6.04E–07 | 1.19E–04 |
|  | *Hsph1* | 2.232472 | 0.243805 | 9.74E–07 | 1.81E–04 |
|  | *Necab2* | 2.583777 | 0.298136 | 2.08E–06 | 3.39E–04 |
|  | *Mgat2* | 2.804783 | 0.274240 | 2.49E–06 | 3.98E–04 |
|  | *Dcaf12l1* | 2.923632 | 0.271993 | 2.67E–06 | 4.18E–04 |
|  | *Srsf2* | 3.097662 | 0.280200 | 2.83E–06 | 4.30E–04 |
|  | *Bag1* | 1.657995 | 0.215308 | 4.52E–06 | 6.24E–04 |
|  | *Gpd2* | 3.433416 | 0.243455 | 6.14E–06 | 7.87E–04 |
|  | *Pde3b* | 2.027260 | 0.200242 | 6.96E–06 | 8.75E–04 |
|  | *Gc* | 1.795676 | 0.256336 | 7.29E–06 | 8.96E–04 |
|  | *Gpx3* | 1.992830 | 0.261711 | 1.11E–05 | 1.29E–03 |
|  | *Ubxn4* | 11.430235 | 0.210008 | 1.13E–05 | 1.30E–03 |
|  | *Eif3b* | 2.425380 | 0.248706 | 1.31E–05 | 1.47E–03 |
|  | *Syt4* | 5.477879 | 0.257428 | 1.56E–05 | 1.71E–03 |
|  | *Il1r1* | 10.469869 | 0.230564 | 1.68E–05 | 1.79E–03 |
|  | *Ang* | 2.794983 | 0.247319 | 2.32E–05 | 2.37E–03 |
|  | *Mrps12* | 5.711173 | 0.260891 | 2.39E–05 | 2.37E–03 |
|  | *Kcnq1ot1* | 7.313281 | 0.254897 | 2.45E–05 | 2.39E–03 |
|  | *Pdyn* | 1.851114 | 0.271729 | 2.54E–05 | 2.45E–03 |
|  | *Itpr2* | 1.834198 | 0.221274 | 3.09E–05 | 2.90E–03 |

**Table S5 Sex-independent T2D altered genes (continued)**

| **Type** | **Gene symbol** | **BaseMean** | **Log_2_ fold change** | ***P* value** | ***P* adjust** |
| --- | --- | --- | --- | --- | --- |
| Up-regulated genes | *Map7d2* | 1.918495 | 0.213524 | 3.45E–05 | 3.11E–03 |
|  | *Erp29* | 3.750680 | 0.231476 | 7.68E–05 | 6.26E–03 |
|  | *Hdlbp* | 7.468171 | 0.203833 | 7.87E–05 | 6.34E–03 |
|  | *Atp1a1* | 5.689841 | 0.223233 | 8.48E–05 | 6.71E–03 |
|  | *Tagln2* | 3.836652 | 0.235306 | 1.05E–04 | 8.08E–03 |
|  | *Chchd10* | 5.005076 | 0.223993 | 1.12E–04 | 8.47E–03 |
|  | *Rpl23* | 21.835487 | 0.215808 | 1.82E–04 | 1.25E–02 |
|  | *Rpsa* | 9.170266 | 0.227070 | 1.88E–04 | 1.27E–02 |
|  | *Luc7l2* | 3.477278 | 0.250265 | 1.95E–04 | 1.30E–02 |
|  | *Fxyd6* | 3.534887 | 0.214029 | 3.88E–04 | 2.29E–02 |
|  | *C1qa* | 2.489959 | 0.233978 | 4.66E–04 | 2.64E–02 |
|  | *Isg20* | 10.104787 | 0.241624 | 5.72E–04 | 3.10E–02 |
|  | *Purb* | 3.646563 | 0.205792 | 5.83E–04 | 3.10E–02 |
|  | *Itpa* | 2.710557 | 0.210362 | 8.45E–04 | 4.13E–02 |
|  | *Cdk11b* | 4.502677 | 0.229556 | 9.44E–04 | 4.47E–02 |
|  | *Psmd7* | 3.841401 | 0.225220 | 1.02E–03 | 4.67E–02 |
| Down-regulated genes | *mt–Rnr1* | 116.945950 | –0.248752 | 9.87E–24 | 4.49E–20 |
|  | *Matn2* | 1.401204 | –0.318970 | 7.02E–18 | 2.13E–14 |
|  | *Slc30a8* | 54.602588 | –0.242440 | 1.93E–15 | 3.95E–12 |
|  | *Cox6a2* | 4.163953 | –0.467249 | 5.28E–13 | 6.86E–10 |
|  | *mt–Cytb* | 78.209799 | –0.212301 | 1.91E–12 | 1.93E–09 |
|  | *Cirbp* | 5.099750 | –0.356116 | 3.43E–11 | 2.40E–08 |
|  | *Fmo1* | 1.554349 | –0.290741 | 8.64E–11 | 5.24E–08 |
|  | *Gnai2* | 20.506412 | –0.248437 | 1.57E–10 | 8.95E–08 |
|  | *Pdia3* | 28.154852 | –0.211874 | 1.80E–10 | 9.62E–08 |
|  | *Fos* | 2.163746 | –0.429555 | 2.04E–10 | 1.03E–07 |
|  | *Slc2a2* | 11.776218 | –0.317095 | 4.25E–10 | 2.04E–07 |
|  | *Btg2* | 3.198182 | –0.364822 | 6.38E–10 | 2.90E–07 |
|  | *Dusp1* | 1.506455 | –0.287646 | 4.84E–08 | 1.38E–05 |
|  | *Rps19* | 11.706875 | –0.227914 | 2.32E–07 | 5.15E–05 |
|  | *Fkbp1b* | 5.854021 | –0.329495 | 6.12E–07 | 1.19E–04 |
|  | *Creg1* | 8.935046 | –0.266559 | 7.12E–07 | 1.35E–04 |
|  | *Nipal3* | 7.379032 | –0.227400 | 1.02E–06 | 1.86E–04 |
|  | *Ndufs2* | 6.997309 | –0.315881 | 2.03E–06 | 3.35E–04 |
|  | *Gmpr* | 12.351981 | –0.217912 | 3.15E–06 | 4.69E–04 |
|  | *Tmem215* | 5.878899 | –0.255269 | 4.11E–06 | 5.93E–04 |
|  | *Calm2* | 5.734616 | –0.236913 | 4.28E–06 | 6.08E–04 |
|  | *Ncoa1* | 5.577167 | –0.305769 | 5.08E–06 | 6.90E–04 |
|  | *Tmem181b–ps* | 3.610974 | –0.227967 | 5.16E–06 | 6.91E–04 |
|  | *Swi5* | 9.617502 | –0.231452 | 5.34E–06 | 7.05E–04 |
|  | *Ier2* | 1.352050 | –0.224118 | 7.02E–06 | 8.75E–04 |
|  | *Mt2* | 4.879097 | –0.343791 | 8.13E–06 | 9.73E–04 |

**Table S5 Sex-independent T2D altered genes (continued)**

| **Type** | **Gene symbol** | **BaseMean** | **Log2 fold change** | ***P* value** | ***P* adjust** |
| --- | --- | --- | --- | --- | --- |
| Down-regulated genes | *Sphkap* | 9.463111 | –0.266648 | 1.59E–05 | 1.72E–03 |
|  | *Mt1* | 12.636225 | –0.300537 | 1.90E–05 | 2.00E–03 |
|  | *H1f0* | 3.174846 | –0.255071 | 2.04E–05 | 2.14E–03 |
|  | *Sfrp5* | 2.012791 | –0.335367 | 3.01E–05 | 2.85E–03 |
|  | *Cars* | 1.800792 | –0.210015 | 3.42E–05 | 3.11E–03 |
|  | *Pcx* | 2.284458 | –0.213215 | 8.25E–05 | 6.58E–03 |
|  | *Fam210b* | 2.596242 | –0.216901 | 1.13E–04 | 8.52E–03 |
|  | *Cd81* | 2.098660 | –0.273120 | 1.51E–04 | 1.08E–02 |
|  | *A330076H08Rik* | 6.045424 | –0.204937 | 1.81E–04 | 1.25E–02 |
|  | *Taok2* | 2.592324 | –0.223456 | 2.18E–04 | 1.43E–02 |
|  | *Gjd2* | 2.912164 | –0.217764 | 3.11E–04 | 1.89E–02 |
|  | *Nagk* | 3.313107 | –0.219245 | 5.20E–04 | 2.87E–02 |
|  | *Jun* | 1.971693 | –0.232372 | 6.89E–04 | 3.48E–02 |
|  | *Mthfd2* | 2.600966 | –0.208770 | 9.90E–04 | 4.62E–02 |
