# Supplementary material for: Single-cell RNA Sequencing Reveals Sexually Dimorphic Transcriptome and Type 2 Diabetes Genes in Mouse Islet β Cells
Source: Genomics Proteomics Bioinformatics. 2021 Sep 24;19(3):408–22. doi: 10.1016/j.gpb.2021.07.004 (PMC8864195; doi:10.1016/j.gpb.2021.07.004)
Supplement: Supplementary Table S6 [file mmc7.docx]

**Table S6 Sex-dependent T2D altered genes**

| **Group** | **Gene symbol** | **BaseMean** | **Log_2_ fold change** | **Ci.lo** | **Ci.hi** | ***P* value** | ***P* adjust** |
| --- | --- | --- | --- | --- | --- | --- | --- |
| Female-dependent T2D up-regulated genes | *Fxyd2* | 1.21754 | 0.47391 | 0.35668 | 0.59114 | 7.43E–14 | 3.32E–10 |
|  | *Cpe* | 42.79065 | 0.37275 | 0.26913 | 0.47637 | 3.33E–11 | 4.00E–08 |
|  | *Serp1* | 17.10865 | 0.40687 | 0.28720 | 0.52654 | 4.14E–10 | 3.36E–07 |
|  | *Scg3* | 36.30745 | 0.26298 | 0.17760 | 0.34837 | 1.82E–08 | 1.11E–05 |
|  | *Cd9* | 1.16846 | 0.25612 | 0.17250 | 0.33975 | 2.25E–08 | 1.26E–05 |
|  | *Igfbp7* | 1.25347 | 0.24994 | 0.16432 | 0.33556 | 1.10E–07 | 5.15E–05 |
|  | *Ttr* | 44.79182 | 0.34848 | 0.22289 | 0.47408 | 4.98E–07 | 2.02E–04 |
|  | *Rnase4* | 18.95756 | 0.37715 | 0.23692 | 0.51738 | 1.18E–06 | 4.23E–04 |
|  | *Stip1* | 2.44458 | 0.34322 | 0.20726 | 0.47917 | 5.89E–06 | 1.55E–03 |
|  | *Ero1lb* | 45.05701 | 0.27708 | 0.16455 | 0.38961 | 1.04E–05 | 2.52E–03 |
|  | *Pnrc1* | 1.79108 | 0.33538 | 0.19337 | 0.47739 | 2.60E–05 | 5.14E–03 |
|  | *Tmem160* | 5.71248 | 0.38975 | 0.22358 | 0.55592 | 2.99E–05 | 5.69E–03 |
|  | *Etv1* | 1.27580 | 0.22790 | 0.13023 | 0.32558 | 3.33E–05 | 5.84E–03 |
|  | *Cmas* | 1.50019 | 0.27776 | 0.15642 | 0.39910 | 4.89E–05 | 8.24E–03 |
|  | *Aplp1* | 9.90697 | 0.33972 | 0.18500 | 0.49445 | 1.07E–04 | 1.43E–02 |
|  | *Pcsk1n* | 62.37328 | 0.21404 | 0.11624 | 0.31185 | 1.13E–04 | 1.48E–02 |
|  | *Akap13* | 3.94333 | 0.34797 | 0.18894 | 0.50701 | 1.14E–04 | 1.48E–02 |
|  | *Manf* | 20.67518 | 0.27022 | 0.14384 | 0.39659 | 1.71E–04 | 1.88E–02 |
|  | *Vcp* | 10.24876 | 0.29427 | 0.15582 | 0.43271 | 1.89E–04 | 1.99E–02 |
|  | *Mbnl2* | 4.20717 | 0.28883 | 0.15244 | 0.42522 | 2.02E–04 | 2.07E–02 |
|  | *Senp5* | 1.44107 | 0.21588 | 0.11341 | 0.31836 | 2.20E–04 | 2.18E–02 |
|  | *Depp1* | 1.38599 | 0.23469 | 0.11784 | 0.35155 | 4.70E–04 | 4.20E–02 |
| Female-dependent T2D down-regulated genes | *Shisal2b* | 1.92446 | –0.41306 | –0.57659 | –0.24952 | 5.81E–06 | 1.55E–03 |
|  | *Chac1* | 1.63104 | –0.34533 | –0.48597 | –0.20468 | 1.12E–05 | 2.56E–03 |
|  | *Rpl37a* | 15.83230 | –0.23590 | –0.34112 | –0.13068 | 7.26E–05 | 1.10E–02 |
|  | *Fkbp9* | 3.99751 | –0.29107 | –0.42565 | –0.15649 | 1.40E–04 | 1.77E–02 |
|  | *Mid1ip1* | 2.02275 | –0.28622 | –0.41870 | –0.15375 | 1.43E–04 | 1.77E–02 |
|  | *Scn9a* | 1.45012 | –0.22446 | –0.32970 | –0.11921 | 1.79E–04 | 1.93E–02 |
|  | *Rps24* | 6.19606 | –0.27068 | –0.39926 | –0.14211 | 2.22E–04 | 2.18E–02 |
|  | *Rps18* | 10.22331 | –0.23190 | –0.34636 | –0.11743 | 4.11E–04 | 3.71E–02 |
|  | *Syp* | 4.85908 | –0.26878 | –0.40404 | –0.13353 | 5.51E–04 | 4.78E–02 |
| Male-dependent T2D up-regulated genes | *Iapp* | 1895.74737 | 0.30829 | 0.26272 | 0.35387 | 2.56E–36 | 2.35E–32 |
|  | *Trpm5* | 2.97837 | 0.39723 | 0.24743 | 0.54704 | 1.65E–06 | 3.37E–04 |
|  | *Prkcb* | 3.37772 | 0.31870 | 0.19290 | 0.44451 | 5.20E–06 | 9.75E–04 |
|  | *Gmds* | 2.24646 | 0.31564 | 0.18936 | 0.44193 | 7.16E–06 | 1.29E–03 |
|  | *Nf1* | 1.34919 | 0.22416 | 0.13181 | 0.31651 | 1.40E–05 | 2.21E–03 |
|  | *Arglu1* | 1.61286 | 0.24156 | 0.14108 | 0.34205 | 1.72E–05 | 2.60E–03 |
|  | *Gnb1* | 2.70566 | 0.31763 | 0.18361 | 0.45164 | 2.34E–05 | 3.35E–03 |
|  | *Tmed3* | 16.78187 | 0.21868 | 0.12268 | 0.31467 | 5.21E–05 | 6.30E–03 |
|  | *Rps13* | 6.03796 | 0.28278 | 0.15675 | 0.40881 | 7.00E–05 | 8.34E–03 |

**Table S6 Sex-dependent T2D altered genes (continued)**

| **Group** | **Gene symbol** | **BaseMean** | **Log_2_ fold change** | **Ci.lo** | **Ci.hi** | ***P* value** | ***P* adjust** |
| --- | --- | --- | --- | --- | --- | --- | --- |
| Male-dependent T2D up-regulated genes | *9530091C08Rik* | 2.04870 | 0.27914 | 0.15327 | 0.40500 | 8.71E–05 | 9.75E–03 |
|  | *Sh3pxd2a* | 3.90795 | 0.32470 | 0.17773 | 0.47166 | 9.34E–05 | 9.97E–03 |
|  | *Rpl8* | 15.78232 | 0.24476 | 0.12725 | 0.36227 | 2.60E–04 | 2.09E–02 |
|  | *Rps27a* | 9.81674 | 0.22991 | 0.11757 | 0.34225 | 3.45E–04 | 2.62E–02 |
|  | *Rpl36al* | 5.52900 | 0.21809 | 0.11152 | 0.32466 | 3.45E–04 | 2.62E–02 |
|  | *Tmem63b* | 1.65110 | 0.21515 | 0.10775 | 0.32254 | 4.80E–04 | 3.27E–02 |
|  | *Rpl12* | 2.90299 | 0.25296 | 0.12555 | 0.38038 | 5.50E–04 | 3.64E–02 |
|  | *Fmn2* | 1.91945 | 0.24689 | 0.12061 | 0.37316 | 6.89E–04 | 4.28E–02 |
|  | *Rpl7a* | 2.58286 | 0.22402 | 0.10879 | 0.33925 | 7.48E–04 | 4.58E–02 |
| Male-dependent T2D down-regulated genes | *Cox4i1* | 75.04082 | –0.29021 | –0.35570 | –0.22471 | 1.50E–16 | 2.75E–13 |
|  | *Malat1* | 1398.58100 | –0.21492 | –0.26458 | –0.16526 | 6.31E–16 | 9.66E–13 |
|  | *Ndufb7* | 15.13873 | –0.30590 | –0.40290 | –0.20891 | 7.22E–09 | 3.32E–06 |
|  | *Ucn3* | 59.20021 | –0.26270 | –0.35005 | –0.17534 | 3.84E–08 | 1.47E–05 |
|  | *Ubc* | 22.89224 | –0.24426 | –0.33358 | –0.15494 | 7.11E–07 | 1.76E–04 |
|  | *Pura* | 13.02289 | –0.26435 | –0.37381 | –0.15489 | 1.56E–05 | 2.38E–03 |
|  | *Ivd* | 2.45318 | –0.32256 | –0.45906 | –0.18605 | 2.49E–05 | 3.46E–03 |
|  | *Gnmt* | 1.55539 | –0.25674 | –0.36876 | –0.14473 | 4.64E–05 | 5.68E–03 |
|  | *Selenow* | 6.00877 | –0.27767 | –0.40718 | –0.14815 | 1.60E–04 | 1.48E–02 |
|  | *Zfyve27* | 1.54321 | –0.20914 | –0.30691 | –0.11137 | 1.66E–04 | 1.51E–02 |
|  | *H3f3b* | 8.30794 | –0.24870 | –0.36533 | –0.13207 | 1.75E–04 | 1.55E–02 |
|  | *Cct7* | 5.03374 | –0.25950 | –0.38476 | –0.13424 | 2.84E–04 | 2.27E–02 |
|  | *Cdk12* | 3.99929 | –0.29476 | –0.43965 | –0.14988 | 3.79E–04 | 2.78E–02 |

*Note:* Ci.lo, low bound of confidence interval; Ci.hi, high bound of confidence interval.
